# Activated Endolysosomal Cation Channel TRPML1 Facilitates Maturation of α-Synuclein-Containing Autophagosomes

**DOI:** 10.3389/fncel.2022.861202

**Published:** 2022-07-06

**Authors:** Maike R. Pollmanns, Judith Beer, Ines Rosignol, Natalia Rodriguez-Muela, Björn H. Falkenburger, Elisabeth Dinter

**Affiliations:** ^1^Department of Neurology, RWTH University Aachen, Aachen, Germany; ^2^Department of Neurology, University Hospital Carl Gustav Carus, Technische Universität Dresden, Dresden, Germany; ^3^Deutsches Zentrum für Neurodegenerative Erkrankungen (DZNE), Dresden, Germany; ^4^Center for Regenerative Therapies Dresden (CRTD), Dresden, Germany; ^5^Max Planck Institute for Molecular Cell Biology and Genetics, Dresden, Germany; ^6^JARA-Institute Molecular Neuroscience and Neuroimaging, Forschungsszentrum Jülich GmbH and RWTH Aachen University, Aachen, Germany

**Keywords:** Parkinson’s disease, synuclein, TRPML1, mucolipin-1, ML-SA1, autophagy, autolysosome maturation, acidification

## Abstract

**Background**: Protein aggregates are degraded *via* the autophagy-lysosome pathway and alterations in the lysosomal system leading to the accumulation of pathogenic proteins, including aggregates of α-synuclein in Parkinson’s disease (PD). The importance of the endolysosomal transient receptor potential cation channel, mucolipin subfamily 1 (TRPML1) for the lysosomal function is highlighted by the fact that TRPML1 mutations cause the lysosomal storage disease mucolipidosis type IV. In this study, we investigated the mechanism by which activation of TRPML1 affects the degradation of α-synuclein.

**Methods**: As a model of α-synuclein pathology, we expressed the pathogenic A53Tα-synuclein mutant in HEK293T cells. These cells were treated with the synthetic TRPML1 agonist ML-SA1. The amount of α-synuclein protein was determined by immunoblots. The abundance of aggregates and autolysosomal vesicles was determined by fluorescence microscopy and immunocytochemistry. Findings were confirmed by life-cell imaging and by application of ML-SA1 and the TRPML1 antagonist ML-SI3 to human dopaminergic neurons and human stem cell-derived neurons.

**Results**: ML-SA1 reduced the percentage of HEK293T cells with α-synuclein aggregates and the amount of α-synuclein protein. The effect of ML-SA1 was blocked by pharmacological and genetic inhibition of autophagy. Consistent with TRPML function, it required the membrane lipid PI(3,5)P_2,_ and cytosolic calcium. ML-SA1 shifted the composition of autophagosomes towards a higher fraction of mature autolysosomes, also in presence of α-synuclein. In neurons, inhibition of TRPML1 by its antagonist ML-SI3 blocked autophagosomal clearance, whereas the agonist ML-SA1 shifted the composition of a-synuclein particles towards a higher fraction of acidified particles. ML-SA1 was able to override the effect of Bafilomycin A1, which blocks the fusion of the autophagosome and lysosome and its acidification.

**Conclusion**: These findings suggest, that activating TRPML1 with ML-SA1 facilitates clearance of α-synuclein aggregates primarily by affecting the late steps of the autophagy, i.e., by promoting autophagosome maturation. In agreement with recent work by others, our findings indicate that TRPML1 might constitute a plausible therapeutic target for PD, that warrants further validation in rodent models of α-synuclein pathology.

## Introduction

Parkinson’s disease (PD) is a neurodegenerative disease characterized by cytoplasmic aggregates of the protein α-synuclein. Rare familial forms can be caused by point mutations in the α-synuclein gene including the A53T-mutation (Polymeropoulos et al., 1997; Guella et al., 2016). Mutations and increased expression of α-synuclein cause enhanced aggregation. Accordingly, polymorphisms in the α-synuclein locus are a risk factor for sporadic PD. The pathological hallmark of PD, Lewy bodies, consists of large clusters of α-synuclein aggregates (Spillantini et al., 1997; Tanaka et al., 2004).

Cells have several mechanisms to remove aggregated and misfolded proteins. Among these, the autophagy-lysosome pathway is recruited with an increasing α-synuclein burden (Ebrahimi-Fakhari et al., 2011). In macroautophagy (henceforth referred to as autophagy), pieces of cytosol containing e.g., aggregated proteins, are engulfed by a membrane, forming double membrane vesicles (autophagosomes) that fuse with lysosomes to degrade their content (Nakatogawa et al., 2009; Johansen and Lamark, 2011). Promoting autophagy is seen as a plausible strategy to remove α-synuclein aggregates and stop disease progression in PD. Two main strategies have been pursued to promote autophagy. One strategy has been to increase the formation of new autophagosomes. Autophagy initiation is regulated by 5’-AMP-activated protein kinase (AMPK), the mammalian target of rapamycin complex 1 (mTORC1), and their downstream signaling cascade (Boland et al., 2018). Accordingly, autophagy can be increased by drugs that induce AMPK such as metformin or inhibit mTORC1 such as rapamycin, but also ABL1 inactivators such as nilotinib or sirtuin 1 inducers such as nicotinamide (Boland et al., 2018). A second strategy has been to facilitate autophagosome maturation, which includes autophagosome fusion with lysosomes, autolysosome acidification, and enzymatic degradation of their content. The small Rab-GTPase Rab7 is a regulator for autolysosome fusion. We recently showed that Rab7 reduces α-synuclein pathology in the cell, fly, and rodent models of PD by acting through its downstream effector Fyco1 (Dinter et al., 2016; Saridaki et al., 2018; Szegö et al., 2022).

Overexpression of α-synuclein impairs autophagy (Winslow et al., 2010) and mutations of genes in the ALP lead to familial and early onset forms of PD (Dehay et al., 2012; Friedman et al., 2012; Koyano et al., 2014). For instance, mutations in the GBA gene, encoding the lysosomal enzyme glucocerebrosidase are the most common genetic risk factor for PD and underlie the lysosomal storage disorder Gaucher’s disease (Sidransky et al., 2009). Currently investigated therapeutic strategies addressing late steps in autophagy include the administration of ambroxol, a chaperone for the lysosomal enzyme glucocerebrosidase (Magalhaes et al., 2018). These findings demonstrate that there are shared mechanisms of cellular dysfunction in neurodegenerative disorders and lysosomal storage diseases. In both, impairment of the autophagy-lysosome pathway leads to the accumulation of proteins and lipids, dysfunctional mitochondria, oxidative stress, and apoptosis (Junn and Mouradian, 2002; Smith et al., 2005; Kiselyov et al., 2007; Szego et al., 2019).

Mucolipidosis type IV (MLIV) is a lysosomal storage disorder (Lieberman et al., 2012) caused by loss-of-function mutations in the MCOLN1 gene encoding the endolysosomal non-selective cation channel TRPML1 (Bargal et al., 2000; Bassi et al., 2000). TRPML1 has been implicated in calcium signaling during the fusion of lysosomes with mitochondrial, endosomal, and autophagosomal membranes (LaPlante et al., 2006; Cheng et al., 2010; Abe and Puertollano, 2011; Wong et al., 2012; Peng et al., 2020). TRPML requires the signaling lipid phosphatidylinositol-3,5-bisphosphate (PI(3,5)P_2_; Dong et al., 2010; McCartney et al., 2014). TRPML1 activation has recently been shown to restore deficient lysosomal exocytosis of α-synuclein in neurons with loss-of-function mutation in lysosomal ATP13A2 (Tsunemi et al., 2019). Flies lacking the TRPML1 homolog showed incomplete autophagy (Wong et al., 2012). Accordingly, autophagosome turnover is defective in fibroblasts derived from patients with mucolipidosis type IV, leading to an accumulation of autophagosomes (Vergarajauregui et al., 2008; Curcio-Morelli et al., 2010).

These findings indicate that activating TRPML1 could be a promising strategy to induce autophagy and promote the clearance of α-synuclein aggregates. To confirm this hypothesis, we used the cell permeable Mucolipin-synthetic agonist (ML-SA1) to specifically and potently activate TRPML-1 (Shen et al., 2012), and the mucolipin-synthetic antagonist (ML-SI3) to block TRPML1 (Leser et al., 2021). We expressed the disease-related A53T mutant of α-synuclein in HEK293T cells and determined whether treatment with ML-SA1 affects the amount of A53T-α-synuclein protein. Given that TRPML1 activation has been shown to both activate autophagosome biogenesis and promote autophagosome fusion/maturation, we analyzed in detail the composition of synuclein-containing autophago-lysosomal vesicles and their subcellular localization to identify the molecular and cellular mechanisms by which ML-SA1 produces its effect. Findings were validated using live-cell imaging and human neurons.

## Material and Methods

### HEK293T Cell Culture and Transfection

HEK293T (RRID:CVCL_0063) cells were cultured in Dulbecco’s modified Eagle’s medium (DMEM) supplemented with 10% fetal calf serum. By analysis of 21 genetic loci the cells were validated in November 2017 (Promega, Power Plex 21 PCR Kit carried out by Eurofins MedigenomixForensiker, Ebersberg, Germany). Cells were plated on poly-L-Lysine coated glass coverslips or on plastic plates. Twenty-four hours after plating, cells were transfected using metafectene (Cat# T020-5.0 Biontex Laboratories, Munich, Germany) following the manufactures instructions.

### hiPSC-Cell Culture

Neurons derived from human-induced pluripotent stem cells (hiPSCs) were cultured and differentiated as described in Reinhardt et al. (2013), starting from a pre-differentiated neuronal progenitor state kindly provided by Jared Sterneckert. In short, progenitors were cultured in N2B27 medium (50:50 DMEM-F12 (Invitrogen, Paisley, UK)/Neurobasal (Invitrogen, Paisley, UK) with 1:200 N2 supplement (Invitrogen, Grand Island, Kentucky, USA), 1:100 B27 supplement lacking vitamin A (Invitrogen, Grand Island, Kentucky, USA) and 1% penicillin/streptomycin/glutamine (Life Technologies, Grand Island, Kentucky, USA). During expansion, N2B27 was freshly supplemented with 3 μM CHIR99021 (Cayman, Ann Arbor, Michigan, USA), 150 μM ascorbic acid (AA) (Sigma-Aldrich, St. Louis, Missouri, USA), and 0.5 μM smoothened agonist (SAG) (Cayman, Ann Arbor, Michigan, USA). For differentiation, 0.8*10^6^ cells were seeded on Matrigel-coated (Corning, New York, USA) 6-well plates (Corning, Kennebunk, Maine, USA) and cultured for 6 days in N2B27 supplemented with 1 ng/ml Brain-derived neurotropic factor (BDNF; PeproTech, Hamburg, Germany), 0.2 mM AA, 1 μM retinoic acid (Sigma-Aldrich, St. Louis, Missouri, USA), 1 ng/ml Glial-derived neurotrophic factor (GDNF; Sigma-Aldrich, St. Louis, Missouri, USA) and 0.5 μM SAG. For final maturation, cells were maintained in N2B27 with 0.1 mM dibutyrylcyclic adenosine monophosphate (Sigma-Aldrich, St. Louis, Missouri, USA), 2 ng/ml BDNF, 0.2 mM AA, 1 ng/ml TGFβ-3 (PeproTech, Hamburg, Germany) and 2 ng/ml GDNF. During the first 2 days of maturation, 5 ng/ml Activin A (Biomol, Hamburg, Germany) was added to the medium. On maturation day 2, cells were split in the final format: 50,000 cells/well, 96-well plates (Greiner bio-one, Kremsmünster, Austria) coated with poly-L-ornithine (Sigma-Aldrich, St. Louis, Missouri, USA) and 1% Laminin (Roche, Mannheim, Germany). One day after the final plating, neurons were transduced with lentiviral vector containing Synuclein-T2A-GFP. Twenty-one days after lentiviral transduction, cells were treated with ML-SA1 or DMSO.

### LUHMES Cell Culture

LUHMES cells were cultured and differentiated as described before (Scholz et al., 2011; Neuhof et al., 2021). In short, Cells were cultured in culture dishes (Nunclon Delta EasY, Thermofisher, Waltham, Massachusetts, USA), precoated with 50 μg/ml Poly-L-ornithine (Sigma-Aldrich, St. Louis, Missouri, USA) and 1 μg/ml human plasma fibronectin (Gibco, Thermo Fisher Scientific, Waltham, USA) in distilled water at 37°C for 3 h. Undifferentiated LUHMES cells were cultured in Dulbecco’s modified Eagle’s medium/F12 (DMEM/F12, Gibco, USA), supplemented with 1× N-2 supplement (Gibco/Invitrogen, Thermo Fisher Scientific, Waltham, USA), L-Glutamin (Gibco/Invitrogen, USA) and 40 ng/ml human recombinant basic FGF (bFGF, Sigma-Aldrich, USA), at 37°C in 5% CO_2_ incubator.

Cell differentiation was done as described before (Scholz et al., 2011; Neuhof et al., 2021). Briefly, 0.6× 10^6^ LUHMES cells were seeded onto a pre-coated T25 flask. 24 h later, medium change was performed with a differentiation medium containing DMEM/F12, N-2 supplement, L-Glutamine, 1 μg/ml tetracycline (Sigma-Aldrich, USA), 40 ng/ml human recombinant GDNF (PeproTech Germany, Hamburg, Germany) and 1 mmol/L dibutyryl cAMP (Sigma-Aldrich, USA). After 2 days of differentiation, cells were re-plated to pre-coated 96-well plates or 24-well plates and lentiviral transduction was performed 3 h after seeding. Medium change was performed after 24 h following every other day. On day 9 of differentiation, cells were treated with ML-SA1, ML-SI3, BafA1, or DMSO for 2 h or 24 h. Validation of dopaminergic differentiation was performed *via* immunostaining on day 0 and day 9 of differentiation (Supplementary Figures 4A,B).

### Lentivirus Production

We used 3rd-generation lentivirus as described before (Szego et al., 2019) containing full-length human α-synuclein cDNA on vector CD526A-1 followed by a T2A-GFP sequence under the vector promotor EF1α. For virus production a second-generation lentivirus packaging system was used, based on the plasmids pcziVSV-G and pCD/NL-BH, kindly provided by Prof. Dr. Dirk Lindemann. For the production of viral particles, HEK293T cells were plated in 10 cm dishes (Greiner bio-one, Kremsmünster, Austria) and transfected with both packaging plasmids and the corresponding transfer vector using PEI (Sigma-Aldrich, St. Louis, Missouri, USA). Viral supernatant was collected 48 h after transfection and concentrated by ultracentrifugation. The resulting viral particles were used to transduce mature neurons. To adjust neuronal infection efficiencies, viral particle infectious units were calculated by fluorescence microscopy of infected HEK293T cells. Experiments with lentivirus were performed to appropriate safety regulations in TU Dresden with the permit Az. 45-8452/120.

### Chemical Treatments

Unless noted otherwise, HEK293T cells were chemically treated 6 h after transfection for 18 h and subsequently processed and analyzed.

ML-SA1 (Sigma-Aldrich Chemie GmbH, Taufkirchen, Germany) was dissolved in DMSO to a 25 mM stock solution. It was used in a final concentration of 25 μM for HEK cells and depicted concentrations for neurons. ML-SI3 (MedKoo Biosciences, Inc., Morrisville, USA) was dissolved in DMSO to a 25 mM stock solution using a final concentration on neurons as depicted. Inhibition of fusion of autophagosomes and lysosomes was achieved by incubation with 5 nM bafilomycin A1 (BafA1; Tocris Bioscience, Bristol, GB, stock solution of 1 μM in DMSO). For time-lapse imaging and Western blot analysis, 100 nM bafilomycin A1 was used. Blocking the formation of autophagosomes was achieved by incubation with 2 mM 3-methlyadenine (3-MA; Sigma-Aldrich Chemie GmbH, Taufkirchen, Germany, stock solution 2M in DMSO). The selective Ca^2+^ chelator BAPTA-AM was used in concentrations of 0.1 μM up to 100 μM (Sigma-Aldrich Chemie GmbH, Taufkirchen, Germany, stock solution of 100 mM in DMSO). For chemically induced dimerization, 100 nM rapamycin (Sigma-Aldrich Chemie GmbH, Taufkirchen, Germany) was used for 1 h before processing the cells. Activation of autophagy by rapamycin was achieved by incubation with 100 nM rapamycin for 18 h, 6 h after transfection. The PIKfyve-Inhibitor YM201636 was used in a concentration of 800 nM (Cayman Chemical Company, Michigan, US, stock solution 80 μM in DMSO). For all drugs dissolved in DMSO, an equal concentration of DMSO (Sigma-Aldrich Chemie GmbH, Taufkirchen, Germany) was used as vehicle control.

### Plasmids

A53T-α-synuclein tagged with EGFP by the interaction of a PDZ-binding motif with its PDZ domain was described previously (Opazo et al., 2008). A tandem-fluorescence version (mRFP-GFP) was used as described (Dinter et al., 2016). For the discrimination of cytosolic aggregates from vesicles by chemically-induced dimerization, we used A53T-α-synuclein fused to FRB (FKBP-rapamycin binding domain) and mCherry and in addition FK-506-binding protein (FKBP) fused to GFP (Dinter et al., 2016). ML1N is the cytoplasmic domain of TRPML1, which is described to bind PI(3,5)P_2_ (Li et al., 2013). This plasmid was a generous gift from Haoxing Xu (Department of Molecular, Cellular, and Developmental Biology, University of Michigan, Ann Arbor, USA). Wildtype (WT) Atg5 and a dominant negative (DN) version were used to examine the impact of autophagosome formation. They were obtained from Noboru Mizushimathrough Addgene (#22949 and #22948). To follow particles positive for the autophagosome marker microtubule-associated protein 1A/1B-light chain 3 (LC3) we used RFP-EGFP tandem fluorescence fused to LC3, a gift from Tamotsu Yoshimori (Addgene plasmid #21074) as previously (Hilverling et al., 2021).

### Immunofluorescence Staining

For immunofluorescence staining, neurons and HEK293T cells were fixed with 4% PFA and 5% sucrose, washed twice in PBS and once in 0.1% Triton X in PBS and incubated in blocking solution for 30 min (1% BSA and 0.1% Triton X in PBS). The first antibody against Lamp1 (Abcam ab 24170, rabbit polyclonal, 1:500), MAP2 (Abcam ab5392, chicken polyclonal, 1:2,000), or human α-synuclein (Enzo, ALX-804-258-L001, rat monoclonal, clone 15G7, 1:1,000) was incubated in 0.2% BSA in PBS overnight. For validation of the differentiation of LUHMES primary antibody against Ki-67 (Leica NCL-MM1, mouse, 1:500), Tyrosinhydroxylase (Chemicon, ab152, rabbit, 1:300), VMAT2 (Invitrogen, 48-0900, rabbit, 1:500), Nestin (Chemicon, abd69, rabbit 1:500) and beta III-Tubulin (Millipore, AB9354, chicken 1:500) was used. After washing, the secondary antibody tagged with Cy7 (Genecopeia, Rockville, USA, L144A, 1:600 in 0.2% BSA in PBS) or Alexa-647, Alexa-405, or Alexa-555 (Invitrogen Thermo Fisher Scientific, Waltham, USA, 1:500 in 0.2% BSA in PBS) was incubated at room temperature for 2 h in the dark. Coverslips were mounted on glass slides using Fluoromount-G (Southern Biotech, Birmingham, USA).

### Microscopy of Fixed Cells

For the quantification of EGFP distribution pattern in HEK293T cells, the analysis was carried out as previously described (Dinter et al., 2016). Cells were transfected 24 h after plating on coverslips. Six hours after transfection, cells were chemically treated. Eighteen hours after treatment, cells were washed three times with cold phosphate-buffered saline (PBS) and fixed with paraformaldehyde (4% PFA, 5% sucrose in PBS) for 10 min. Subsequently, coverslips were washed three times in PBS and twice in ddH_2_O. After letting them dry for half an hour, the coverslips were mounted with Fluoromont-G. Using fluorescence microscopy (Olympus BX51 microscope, Olympus, Hamburg, Germany, 40× oil objective), the EGFP distribution pattern was manually classified as homogenous distribution, particles (most likely aggregates), aggresome (large round aggregate), and unhealthy (round, condensed cells). At least 100 cells per coverslip were classified by an investigator blinded for the experimental condition. In each experiment, three coverslips were evaluated per group and the results averaged in mean. “n” corresponds to the number of independent experiments.

For analysis of the vesicle pools in HEK293Tcells, images were acquired with identical acquisition settings for all samples using fluorescence microscopy [Olympus IX81 equipped with 60× oil objective NA 1.3, Hammatsu CCD Camera, and Xcellence Software (Olympus, Hamburg, Germany)].

Images of NPC-derived neurons and LUHMES-derived dopaminergic neurons were acquired with identical acquisition settings using confocal microscopy [Zeiss—Xio Observer.Z1 with inverted stand, 40× air objective, Yokogawa CSU-X1M 5000 dual camera and ZEN Blue 2011 Software (Zeiss, Oberkochen, Germany)]. Images were processed in ImageJ (Version 2.1.0/1.53c) and particle appearance was classified manually blinded for the experimental group. In total, we analyzed 31 NPC-derived neurons and 30 LUHMES-derived dopaminergic neurons for DMSO control and 30 NPC-derived neurons, and 30 LUHMES-derived dopaminergic neurons with ML-SA1 treatment.

### Analysis of Particle Distribution

To determine the position and abundance of particles within HEK293T cells, a “shrink analysis” was used as previously described (Hilverling et al., 2021). Briefly, the cell was outlined as a freehand region of interest (ROI). Using the published custom-written macro the cell outline was progressively eroded by 10% of its original size until only 10% of the original size was left. In each step, the number of particles in the entire cell and the remaining area was quantified.

### Live Cell Imaging

For time-lapse microscopy, cells were grown in 24-well plates with glass bottom (Greiner bio-one, Frickenhausen, Germany) and transfected as described above. One hour before imaging, the medium was changed to Hank’s Buffered Salt Solution with 100 nM BafA1 to induce autophagy by starvation and to block the final steps of autophagy. MLSA1 and DMSO were added directly before starting imaging. We used a Zeiss spinning disk confocal microscope (Zeiss blue software, 40× air objective) equipped with an incubator (37°C, 5% CO_2_) and a motor stage to acquire images at defined positions every 20 min over 80 min. Changes in particle appearance were classified manually based on the resulting images by an investigator blinded for the experimental group. Analysis was done in ImageJ. In total, we analyzed 31 cells for DMSO control and 32 cells with ML-SA1 treatment.

### LDH-Assay

To determine the highest non-toxic concentration of ML-SA1 and ML-SI3 on LUHMES cells, the concentration of extracellular LDH was measured in the medium 24 h after the last medium change with the start of chemical treatment on day 9 of differentiation using the LDH-Cytox Assay Kit (Biolegend, San Diego, California, USA) according to the manufacturer’s instruction. Briefly, for each independent condition, three to four technical replicates were measured. Absorbances were measured at 492 nm; the reference wavelength was 620 nm. Background (medium) absorbance was subtracted from all values, and values were normalized to control cells (DMSO-treated) and to maximal lysed cells (treated with 1% Triton X-100 for 10 min).

### Immunoblot Analysis

Immunoblots for HEK cell lysates were carried out as previously described (Dinter et al., 2016). HEK293T cells were plated in a 6-well plate, transfected, and treated as described above. Twenty-four hours after transfection, cells were washed three times with PBS and lysed for 30 min at 4°C in NP40-lysis buffer (0.5% NP40, 50 mM Tris, 100 mM NaCl, 5 mM MgCl_2_, 1 mM EDTA, pH 8.8) containing protease inhibitor (Pierce, Rockford, IL, USA, Thermo Fisher Scientific, Rockford, USA). The total protein concentration was determined using the DC Protein Assay Kit (Bio-Rad, Munich, Germany). Twenty microgram of protein was subjected to 12% SDS polyacrylamide gel electrophoresis and proteins were blotted onto nitrocellulose membranes. Primary antibodies were incubated overnight at 4°C and HRP-conjugated secondary antibody was incubated for 2 h at room temperature. Detection was carried out by chemiluminescence (SuperSignal West Pico Chemiluminescent Substrate, Thermo Fisher Scientific, Rockford, USA). Bands were quantified using the software BioDocAnalyze (Biometra, Göttingen, Germany) as previously (Dinter et al., 2016). The following antibodies were used: rabbit anti-α-synuclein (1:500, #2642, Cell Signaling Technology, Danvers, USA), rabbit anti-actin (1:1,000, abcam, ab1801, Cambridge, UK), and anti-rabbit IgG (1:10,000, GE Healthcare Life science, Freiburg, Germany).

As described previously, incubation with the α-synuclein antibody in HEK293T lysates showed an unspecific band at around 35 kDa and two bands around 20 kDa. The upper one was only seen in cells transfected with α-synuclein and, therefore, considered as the α-synuclein band (Dinter et al., 2016).

Immunoblot analysis for LUHMES cells and hiPSC-derived neuron lysates was performed as described before (Szego et al., 2019). For protein quantification, cells were lysed in a buffer containing 1% Triton X-100, 25 mM Tris pH 7.5, 150 mM NaCl, 1 mM EDTA, and protease inhibitors. After centrifugation (13,000 *g*, 20 min, 4°C). Twenty microgram of Triton X-100 soluble lysate was loaded onto a 12% Bis-Tris gel for Western blot analysis (NuPAGE, ThermoFisher, USA). Every lysate was run twice in parallel for dry blotting on nitrocellulose and PVDF-membrane in parallel (iBlot, ThermoFisher, USA). After blocking, membranes were incubated with first antibodies against human αSyn (1:4,000, rat, Enzo, ALX-804-258-L001), LC3 (1:2,000, rabbit, Cell Signaling, #2775), p62 (1:5,000, mouse, Abcam, ab56416) and beta-tubulin (1:3,000, rabbit, Cell Signaling, #2146S), HRP-conjugated secondary antibodies were incubated for 1.5 h at room temperature. The signal was visualized with chemiluminescent substrate (SuperSignal West Dura/Femto Chemiluminescent Substrate, Thermo Fisher Scientific, Rockford, USA) and detected with ImageQuant LAS 4000.

### Statistical Analysis

For statistical analysis and presentation of data, GraphPad Prism 7.0 was used (GraphPad Software, La Jolla, USA). Graphs represent mean ± SEM. The test used for comparison in each graph and the number of independent experiments are noted in the figure legend. *p* < 0.05 was considered statistically significant. *p* values are depicted as *p < 0.05, **p < 0.01, ***p < 0.001, ****p < 0.0001. Non-significant differences are depicted as n.s.

## Results

### TRPML-1 Activation by ML-SA1 Reduces α-Synuclein Particles and Protein Amount

To confirm that activating TRPML-1 affects α-synuclein pathology in human cells, we expressed the pathogenic A53T mutant of α-synuclein in HEK293T cells. A53T-α-synuclein was flexibly tagged with EGFP by the interaction of a six amino acid PDZ binding motif added to the C-terminus of α-synuclein, as described (Opazo et al., 2008). Using this technique, aggresomes, aggregates, and vesicular α-synuclein particles can be detected in living and fixed cells. Cells were manually classified regarding their EGFP distribution patterns (Supplementary Figure 1A) cells with a homogenous distribution of EGFP (“homogenous”), cells showing a punctate distribution of EGFP—most likely aggregates (“aggregates”), cells showing round, large aggregates (“aggresome”) and cells with a rounded appearance (“unhealthy”), which suffer apoptosis when followed by time-lapse microscopy. This cell classification assay has been used in previous studies to determine differences between α-synuclein variants and the effects of therapeutic manipulations (Opazo et al., 2008; Karpinar et al., 2009; Krumova et al., 2011; Dinter et al., 2016; Saridaki et al., 2018).

**Figure 1:**
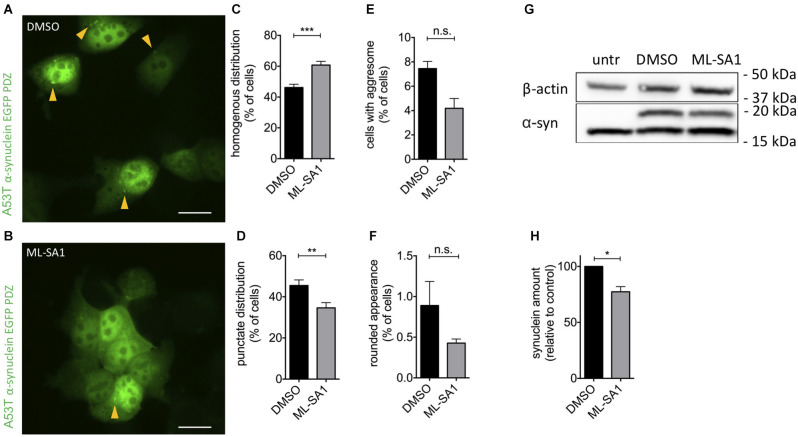
TRPML1-Agonist ML-SA1 reduces α-synuclein aggregates and protein in HEK293T cells. **(A,B)** Example images of HEK293T cells transfected with GFP-tagged A53T-α-synuclein, and treated with 25 μM ML-SA1 or DMSO only as a control, starting 6 h after transfection for 18 h. The scale bar represents 20 μm. **(C–F)** Quantitative analysis of GFP distribution patterns as in Supplementary Figure 1A in about 100 cells per group and experiment, summary of *n* = 3 independent experiments. Two-way ANOVA (factors treatment and phenotype) showed a significant interaction (*p* < 0.0001). Results from Sidak’s multiple comparisons tests are depicted. **(G)** Example immunoblot of lysates from HEK293T cells transfected and treated as in **(A)**. Note that the lower (16 kDa) band is nonspecific since it is present in untransfected cells (Dinter et al., 2016). Full blot shown in Supplementary Figure 1C. **(H)** Quantification of the synuclein band relative to actin in *n* = 3 independent experiments as in **(G)**. Result of unpaired *t*-test depicted with respect to DMSO (*p* = 0.01). *p < 0.05, **p < 0.01, ***p < 0.001, non-significant differences are depicted as n.s.

To activate TRPML1, we used its small molecule activator ML-SA1 in a final concentration of 25 μM. In the control group, 46% of cells showed a homogenous distribution of EGFP, i.e., no number of α-synuclein particles (Figures 1A–C). Application of ML-SA1 increased the number of homogenous cells significantly to 60% and reduced the percentage of cells with punctate distribution (Figures 1B–D). The average number of cells with an aggresome and with round unhealthy appearance were smaller with ML-SA1 treatment, but this difference was not statistically significant—probably due to the small number of cells in these categories (Figures 1E,F).

To determine whether activation of TRPML-1 also reduces the total amount of α-synuclein protein, we performed immunoblot analysis of HEK293T cells transfected and treated as described above. The specific α-synuclein immunoreactivity decreased by 23% under treatment with ML-SA1 compared to the control (Figures 1G,H).

### Effect of ML-SA1 on α-Synuclein Depends on the Signaling Lipid PI(3,5)P_2_

**Figure 2:**
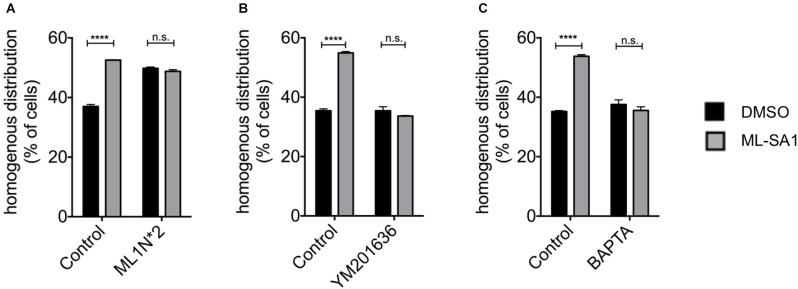
TRPML-1-Agonist ML-SA1 requires Phosphoinositides (PIs) and Ca^2+^. **(A)** HEK293T cells were transfected with GFP-tagged A53T-α-synuclein, and in addition with ML1N*2 (the N-terminus of TRPML1), which binds PI(3,5)P2, or mCherry only as control and treated in addition with 25 μM ML-SA1 or DMSO starting 6 h after transfection for 18 h. GFP distribution patterns were determined in *n* = 3 independent experiments. Only the percentage cells with a homogenous GFP distribution are shown for clarity. Two-way ANOVA (factors transfection and treatment) showed significant interaction (*p* < 0.0001). Results of Sidak’s multiple comparisons test are depicted. **(B)** HEK293T cells were transfected with GFP-tagged A53T-α-synuclein and treated with 800 nM YM201636 or DMSO only as control starting 5 h after transfection and with 25 μM ML-SA1 or DMSO only starting 6 h after transfection. Cells were analyzed 24 h after transfection. GFP distribution pattern was determined. Only the results for homogenous GFP distribution are shown for clarity. Findings are from *n* = 3 independent experiments. Two-way ANOVA (factors YM201636 treatment and ML-SA1 treatment) showed significant interaction (*p* < 0.0001). Results from Sidak’s multiple comparisons tests are depicted. **(C)** HEK293T cells were transfected with GFP-tagged A53T-α-synuclein and treated with 100 nM BAPTA-AM or DMSO only and with 25 μM ML-SA1 or DMSO only starting 6 h after transfection. Cells were analyzed 24 h after transfection. GFP distribution patterns were determined in *n* = 3 independent experiments. Two-way ANOVA (factors BAPTA-AM treatment and ML-SA1 treatment) showed significant interaction (*p* < 0.0001). Results from Sidak’s multiple comparisons tests are depicted. ****p < 0.0001, non-significant differences are depicted as n.s.

Phosphoinositides (PIs) play a key role in various cellular processes involving signaling (Di Paolo and De Camilli, 2006; Ariosa and Klionsky, 2016). Activation of TRPML-1, for instance, requires PI(3,5)P_2_ (Dong et al., 2010; Fine et al., 2018). PI(3,5)P_2_ is generated from PI3P by phosphorylation at the 5-position of the inositol ring by PIKfyve. To confirm that ML-SA1 indeed acts throughTRPML1, we used a protein that binds and sequesters PI(3,5)P_2_, ML1N*2, the N-terminal cytoplasmatic domain of TRPML1 (Dong et al., 2010; Li et al., 2013). We co-expresssed ML1N*2 with A53T-α-synuclein and classified the EGFP distribution pattern upon ML-SA1 treatment. Indeed, coexpression of ML1N*2 blocked the ML-SA1 effect (Figure 2A, only the numbers for cells with homogenous distribution patterns are shown for clarity). Interestingly, expression of ML1N*2 increased the baseline percentage of cells with a homogenous distribution of EGFP.

To verify this finding, we blocked the synthesis of PI(3,5)P_2_ using the PIKfyve inhibitor YM201636. Under control conditions, ML-SA1 increased the percentage of cells with a homogenous distribution. This effect was blocked by treatment with YM201636 (Figure 2B). Together with the effect of ML1N*2, this finding indicates that ML-SA1 indeed acts through the PI(3,5)P_2_-dependent TRPML1 channel.

### ML-SA1 Requires Cytosolic Ca^2+^

TRPML1 is a cation channel and likely mediates the release of Ca^2+^ from lysosomes into the cytosol (Dong et al., 2010). We, therefore, hypothesized that a rise of cytosolic Ca^2+^might mediate the ML-SA1 effect on α-synuclein aggregates. To test this hypothesis, we depleted cytosolic Ca^2+^ using the cell-permanent calcium chelator BAPTA. As Ca^2+^ is essential for many cellular functions, we first determined the highest non-toxic concentration of BAPTA-AM. 100 nM final concentration of BAPTA-AM was associated with a similar number of dead cells compared to untreated control (Supplementary Figure 1B). At this concentration, prolonged exposure to BAPTA-AM did not alter the number of cells with a homogenous distribution of EGFP-tagged A53T-α-synuclein (Figure 2C). The effect of ML-SA1, in contrast, was not observed in the presence of 100 nM BAPTA-AM (Figure 2C). This result indicates that a rise in cytosolic calcium is involved in the effect of ML-SA1 on α-synuclein aggregates.

### ML-SA1 Increases the Number of α-Synuclein Particles Engulfed by a Membrane

**Figure 3:**
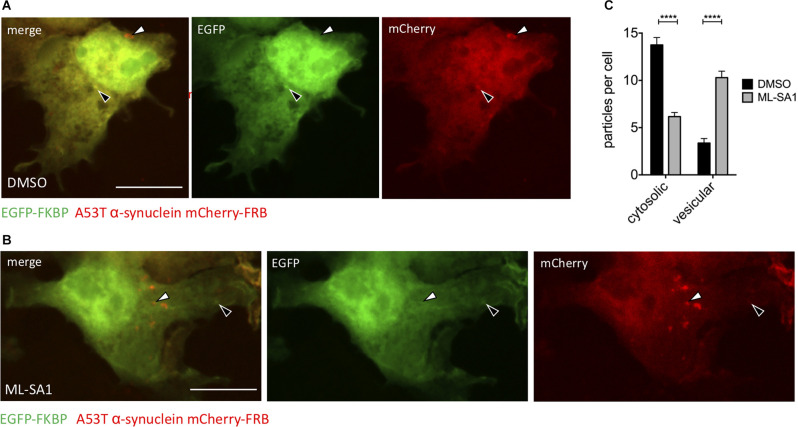
TRPML1-Agonist ML-SA1 increases number of vesicle-bound α-synuclein. **(A,B)** Example images of HEK293T cells transfected with A53T-α-synuclein fused to mCherry-FRB and coexpressed with EGFP-FKBP. Treatment of cells with DMSO **(A)** or 25 μM ML-SA1 for 18 h **(B)**. Addition of rapamycin leads to heterodimerization of FRB and FKBP, recruiting GFP-FKBP to cytosolic synuclein-mCherry-FRB but not to synuclein contained in vesicles. The white arrows mark “mCherry only” particles which represent vesicles, black arrows mark cytosolic α-synuclein particles (yellow in merged images). Scale bars represent 20 μm. **(C)** The number of cytosolic particles and vesicles from *n* = 3 independent experiments. Two-way ANOVA (factors treatment and particle type) showed significant interaction (*p* < 0.0001). Sidak’s multiple comparisons test is depicted with respect to DMSO. ****p < 0.0001, non-significant differences are depicted as n.s.

The ML-SA1 induced decrease of α-synuclein particles and protein levels can be explained by increased clearance. Autophagy—where cytosolic content gets packed into vesicles—is the main degradation pathway of α-synuclein. To discriminate between cytosolic α-synuclein aggregates and α-synuclein aggregates engulfed by a membrane, we used a chemically induced dimerization approach, as described before (Banaszynski et al., 2005; Dinter et al., 2016; Hilverling et al., 2021). We transfected HEK293T cells with the dimerization domain FK506 binding protein (FKBP) fused to EGFP and in addition with mCherry-A53T-α-synuclein fused to the other dimerization domain FKBP-rapamycin binding domain (FRB). Six hours after transfection, cells were treated with ML-SA1 or DMSO for 18 h. One hour before fixation, cells were additionally treated with rapamycin to induce the hetero-dimerization of EGFP-FKBP and A53T-α-synuclein-mCherry-FRB (Figure 3). Colocalization of GFP and mCherry only occurs for cytosolic α-synuclein particles (black arrows in Figures 3A,B) whereas A53T-α-synuclein-mCherry-FRB contained in vesicles is not accessible to EGFP-tagged FKBP (white arrows in Figures 3A,B).

In control cells, around three α-synuclein particles per cell were classified as inside vesicles (Figures 3A–C). Upon ML-SA1 treatment, the number of α-synuclein particles classified as vesicles increased threefold (Figures 3B,C). ML-SA1 thus increased the number of vesicles containing α-synuclein, which most likely represent autophagosomes.

Since TRPML1 can regulate lysosomal mobility, we also analyzed the subcellular location of α-synuclein-positive particles. There was no significant effect of ML-SA1 on the subcellular location of cytosolic particles and vesicles (Supplementary Figures 2A,B).

### The ML-SA1 Effect Requires Autophagy

Given that ML-SA1 increased the number of α-synuclein-containing vesicles—most probably autophagosomes—we took a deeper look into the different steps along the autophagy pathway.

**Figure 4:**
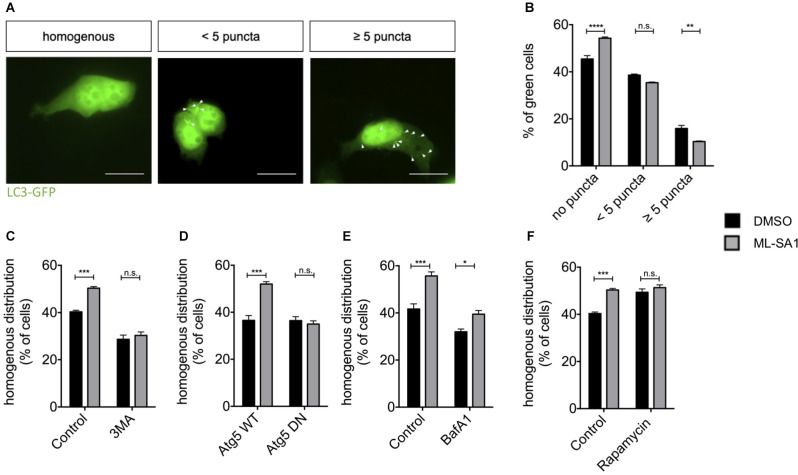
TRPML1-Agonist ML-SA1 promotes autophagy. **(A)** HEK293T cells were transfected with the autophagosome marker LC3-GFP and treated with 25 μM ML-SA1 or DMSO starting 6 h after transfection for 18 h. Example images of possible distribution of LC3 in the cell are shown (white arrows). Scale bars represent 20 μm. **(B)** Quantitative analysis from *n* = 3 independent experiments as described in **(A)** showing the frequency of cells with a homogenous distribution of GFP, cells with less than five puncta, and cells with more than five puncta. Two-way ANOVA (factors phenotype and ML-SA1-treatment) showed a significant interaction (*p* < 0.0001). Sidak’s multiple comparisons test is depicted with respect to DMSO (control). **(C)** HEK293T cells expressing GFP-tagged A53T-α-synuclein were treated with 25 μM ML-SA1, 2 mM 3-MA, both or DMSO only starting 6 h after transfection for 18 h. Results of *n* = 3 independent experiments. Two-way ANOVA (factors 3-MA-treatment and ML-SA1-treatment) showed significant interaction (*p* < 0.0001). Sidak’s multiple comparisons test is depicted with respect to DMSO (control). **(D)** HEK293T cells were transfected with GFP-tagged A53T-α-synuclein and either ATG5 WT or ATG5 DN and treated as described above. Percentage of cells with homogenous GFP distribution from *n* = 3 independent experiments. Two-way ANOVA (factors WT vs. DN Atg5 transfection and ML-SA1-treatment) showed significant interaction (*p* < 0.0001). Sidak’s multiple comparisons test is depicted with respect to DMSO. **(E)** HEK23T cells transfected with GFP-tagged A53T-α-synuclein and treated 6 h after transfection with 25 μM ML-SA1, 5 nM Bafilomycin A1, both or DMSO only for 18 h. Quantitative analysis of *n* = 3 independent experiments is represented. Two-way ANOVA (factors Bafilomycin-A1-treatment and ML-SA1-treatment) showed no significant interaction (*p* = 0.088), in line with significant differences regarding the treatment (ML-SA1: *p* < 0.0001; BafA1: *p* = 0.0002). **(F)** HEK293T cells were transfected with GFP-tagged A53T-α-synuclein and treated with either 25 μM ML-SA1, 100 nM rapamycin, both or DMSO only starting 6 h after transfection for 18 h. Quantitative analysis from *n* = 3 independent experiments showing the frequency of cells with a homogenous distribution of GFP. Two-way ANOVA (factors ML-SA1-treatment and rapamycin-treatment) showed significant interaction (*p* = 0.005). Sidak’s multiple comparisons test is depicted with respect to DMSO (control). *p < 0.05, **p < 0.01, ***p < 0.001, ****p < 0.0001, non-significant differences are depicted as n.s.

To investigate whether activation of TRPML1 by ML-SA1 induces *de novo* formation of autophagosomes in our paradigm, we expressed the autophagosomal marker LC3-EGFP and treated the cells with ML-SA1 or DMSO as a control. Cells were manually classified as cells with a homogenous distribution pattern of LC3-EGFP and cells with LC3-EGFP-positive puncta (Figure 4A). Visible puncta of LC3 are only formed when LC3 accumulates on early autophagosomal membranes; LC3-positive puncta are thus defined as autophagosomes (Pankiv et al., 2007). Interestingly, administration of ML-SA1 for 18 h increased the percentage of cells with a homogenous distribution (Figures 4A,B). The number of cells with few LC3-GFP puncta was not altered by ML-SA1, the number of cells with many puncta was decreased. Scotto Rosato et al. (2019) described the biogenesis of early autophagic vesicles by ML-SA1 and other TRPML1 agonists that could be observed already a few minutes after application. Our finding, therefore, suggests that enhancing the biogenesis of early autophagic vesicles is not the only effect of ML-SA1. Rather, ML-SA1 might also affect the late steps of autophagy, increasing autophagosome maturation and clearance as suggested by the lower number of cells with LC3-tagged autophagosomes (Figure 4B).

In order to test whether the first steps of autophagy are at all required for A53T-α-synuclein particle reduction by ML-SA1, we first used 3-Methyladenine (3-MA), which inhibits autophagy initiation (Blommaart et al., 1997). The reduction of A53T-α-synuclein particles by ML-SA1 was not observed in the presence of 3-MA (Figure 4C). To confirm this finding by genetic manipulation, we expressed WT and DN autophagy-related 5 (Atg5). Atg5 plays a crucial role in the first steps of autophagy, specifically in the formation of the autophagosomal membrane (Hurley and Young, 2017). Indeed, the effect of ML-SA1 treatment was not observed in cells expressing DN Atg5, but in cells expressing WT Atg5 (Figure 4D). Conversely, when autophagy was initiated by treatment with rapamycin, the number of cells without α-synuclein increased, but ML-SA1 had no additional effect (Figure 4F). Taken together, these findings indicate that ML-SA1 affects A53T-α-synuclein particles through autophagy.

Since ML-SA1 might affect the late steps of the autophagy-lysosome pathway, we next tested the effect of Bafilomycin A1 (BafA1), which blocks the fusion of autophagosomes with lysosomes. This effect most probably results from a block of the sarco-endoplasmatic calcium ATPase. In addition, BafA1 inhibits acidification of lysosomes and endosomes by altered H^+^-ATPase activity (Mauvezin and Neufeld, 2015; Klionsky et al., 2016). Treatment with BafA1 decreased the percentage of cells with a homogenous distribution of A53T-α-synuclein tagged with EGFP (black bars in Figure 4E), consistent with clearance of A53T-α-synuclein particles by the autophagy-lysosome pathway. ML-SA1 still showed an effect in cells treated with BafA1 (Figure 4E). This result indicates that ML-SA1 may be able to override the effect of BafA1 on autophagosome-lysosome fusion and/or acidification. Taken together, these findings indicate that ML-SA1 likely acts on α-synuclein at the late steps of the autophagy-lysosome-pathway.

### ML-SA1 Promotes Autolysosomal Maturation

**Figure 5:**
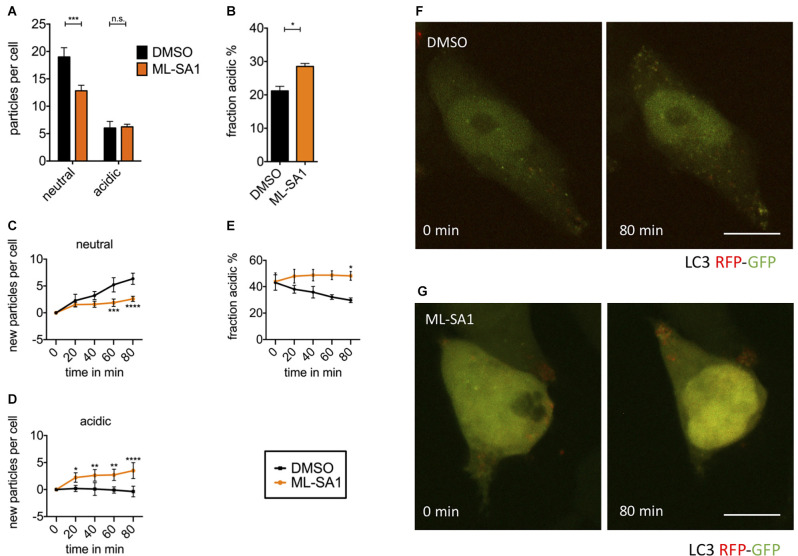
TRPML1- agonist ML-SA1 facilitates autolysosomal maturation. **(A)** HEK293T cells were transfected with the autophagosome marker LC3 tagged with mRFP-EGFP tandem-fluorescence (TFL) and treated for 18 h either with 25 μM ML-SA1 or DMSO. The number of neutral and acidic vesicles from *n* = 3 independent experiments. Two-way ANOVA factors treatment and vesicle type showed significant interaction (*p* < 0.0001). Sidak’s multiple comparisons test is depicted with respect to DMSO (control). **(B)** Fraction of acidic LC3-particles from the experiment described in **(A)**. Result of unpaired *t*-test depicted with respect to DMSO (*p* = 0.01). **(C–F)** Time lapse microscopy: HEK293T cells were transfected with the autophagosome marker LC3-TFL. One hour before, the imaging medium was changed to HBSS containing 100 nM BafA1. Immediately before starting imaging, cells were treated with 25 μM ML-SA1 or DMSO. Imaging was performed every 20 min over 4 h with *n* = 3 experiments. **(C)** Baseline-corrected number of neutral LC3-particles per cell over time in minutes. Two-way ANOVA factors treatment and time showed significant interaction (*p* < 0.0001). Sidak’s multiple comparisons test is depicted with respect to DMSO (control). **(D)** Baseline-corrected number of new acidic LC3-particles per cell over time in minutes. Two-way ANOVA (factors treatment and time) showed significant interaction (*p* < 0.0001). Sidak’s multiple comparisons test is depicted with respect to DMSO (control). **(E)** Fraction of acidic LC3-particles over time in minutes. Two-way ANOVA factors treatment and time showed significant interaction (*p* < 0.0001). Sidak’s multiple comparisons test is depicted with respect to DMSO (control). **(F,G)** Representative images of life-imaged cells as described above, in presence of DMSO **(F)** or ML-SA1 **(G)** at the time of start (0 min) and after 80 min. Scale bars represent 20 μm. *p < 0.05, **p < 0.01, ***p < 0.001,****p < 0.0001, non-significant differences are depicted as n.s.

In order to explore whether ML-SA1 affects the abundance of acidic autophagosomes in general, i.e., without α-synuclein, HEK293T cells were transfected with the autophagosomal marker LC3 tagged with tandem fluorescent tag EGFP-mRFP (TFL) as described before (Dinter et al., 2016; Hilverling et al., 2021). In short, TFL allows a distinction between vesicles with acidic and neutral luminal pH. In neutral pH, GFP and mRFP are functional and appear yellow in merged images. EGFP is quenched by acidic pH, so acidic vesicles appear red in merged images. After ML-SA1 treatment for 24 h, we observed fewer neutral LC3-positive vesicles (neutral/early autophagosomes) and a stable population of acidic LC3-positive vesicles (mature autophagosomes; Figure 5A). The fraction of acidic vesicles was thus increased (Figure 5B). This finding could be explained by ML-SA1 promoting the maturation of autophagosomes. Hence, mature autophagosomes could be cleared during the 24 h incubation.

To observe the immediate effects of ML-SA1 on neutral and acidic autophagosomes, we performed time-lapse imaging of HEK293T cells expressing LC3-TFL. To obtain a sufficient number of autophagosomes for imaging, we induced autophagy by starvation and blocked autophagosome clearance by treatment with BafA1. Consequently, the number of neutral autophagosomes showed a continual increase over the time of recording (Figure 5C black). As expected, the number of mature autolysosomes did not significantly change over time in the presence of BafA1 (Figure 5D black). With MLSA1, the continual increase in neutral autophagosomes over time was significantly less pronounced after 60 min compared to the control condition (Figure 5C orange). This finding is consistent with the data at 24 h obtained without starvation and BafA1 (Figure 5A). It could be explained by the increased maturation of neutral autophagosomes into acidic autophagosomes by ML-SA1. Accordingly, the number of mature autolysosomes increased over time (Figure 5D orange). In total, the percentage of acidic vesicles after 80 min was significantly higher with ML-SA1 compared to control (Figure 5E), again consistent with the findings after 24 h (Figure 5B). This finding suggests that ML-SA1 can partially override the BafA1 effect, consistent with the observation in Figure 4E. The increase in acidic LC3 vesicles was already observed after 20 min, consistent with the rapid onset observed by others (Scotto Rosato et al., 2019).

### ML-SA1 Modifies Vesicle Pools by Facilitating Autophagosome-Lysosome Fusion

**Figure 6:**
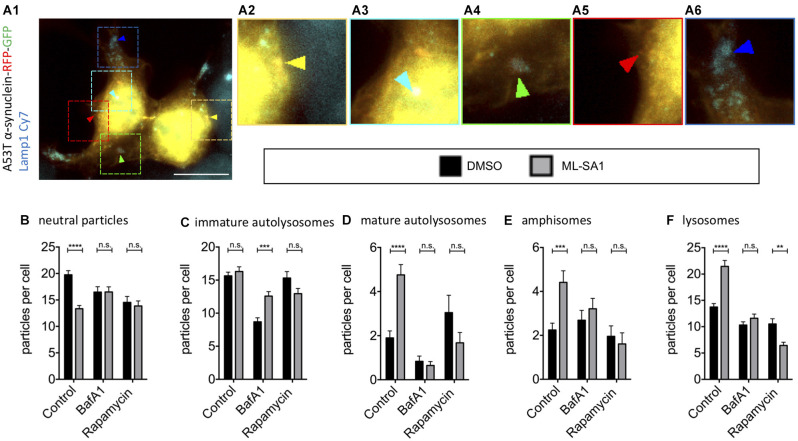
Effect of TRPML1-Agonist ML-SA1 on TFL-tagged A53T-α-synuclein vesicles positive and negative for Lamp1. **(A)** Example image of HEK293T cells transfected with A53T-α-synuclein tagged mRFP-EGFP tandem-fluorescence (TFL) and treated for 18 h either with 25 μM ML-SA1 or with DMSO and additionally 5 nM Bafilomycin A1 or100 nM Rapamycin or DMSO. Cells were further stained against the lysosomal marker Lamp1. The scale bar represents 20 μm. **(A1–6)** Close-up of 15 × 15 μm: yellow arrows mark neutral A53T-α-synuclein particles (neutral particles), light blue arrows mark neutral A53T-α-synuclein/Lamp1-positive vesicles (immature autolysosomes), green arrows mark acidic A53T-α-synuclein/Lamp1-positive vesicles (mature autolysosomes), red arrow marks A53T-α-synuclein/Lamp1-negative acidic vesicles (amphisomes) and blue arrows mark vesicles only positive for LAMP1 (lysosomes). **(B–F)** Abundance and distribution of vesicles from *n* = 3 independent experiments. Vesicles were discriminated by luminal pH and Lamp1 staining as described in **(A)**. Two-way-ANOVA (factors ML-SA1 and BafA1/Rapamycin) showed significant interaction for all vesicle types (values in brackets), **(B)** neutral particles (*p* = 0.0001), **(C)** immature autolysosomes (*p* = 0.001), **(D)** mature autolysosomes (*p* < 0.0001), **(E)** amphisomes (*p* = 0.02), **(F)** lysosomes (*p* < 0.0001). Sidak’s multiple coparisons test is depicted with respect to DMSO. **p < 0.01, ***p < 0.001, ****p < 0.0001, non-significant differences are depicted as n.s.

As the final steps in the autophagy-lysosome pathway might be crucial for the effect of ML-SA1 on α-synuclein, we took a closer look into the effect of ML-SA1 on the different maturation steps during autophagy. Using TFL-tagged α-synuclein and the lysosomal marker Lamp1 as described before (Hilverling et al., 2021), the vesicles involved in these steps can be classified according to their luminal pH and the composition of their membranes. We distinguished five different vesicle types (Figure 6A). ML-SA1 significantly reduced the number of Lamp1-negative neutral α-synuclein particles, which are considered cytosolic aggregates or neutral autophagosomes (Figure 6B). This effect of ML-SA1 was not observed when cells were incubated with BafA1, indicating that fusion of autophagosomes with lysosomes or vesicle acidification is required for the reduction of α-synuclein aggregates and neutral autophagosomes by ML-SA1.

ML-SA1 did not change the abundance of early immature autolysosomes, which are already Lamp1-positive but still neutral vesicles (Figure 6C). BafA1 strongly reduced this population (Figure 6B), consistent with its known inhibition of autophagosome-lysosome fusion. This reduction was partially rescued by ML-SA1, in accordance with the hypothesis that ML-SA1 could partially override the blockade of autophagosome-lysosome fusion by BafA1 noted above (Figures 4E, Figure 5D). ML-SA1 increased the number of mature autolysosomes, which are Lamp1-positive and acidic vesicles (Figure 6D). This effect was blocked by BafA1, suggesting that ML-SA1 might be better at overriding the inhibition of autolysosomal fusion by BafA1 than at rescuing vesicle acidification. ML-SA1 also increased the number of amphisomes, Lamp1-negative acidic vesicles (LNAV; Figure 6E). This effect was also blocked by BafA1. Finally, ML-SA1 increased the number of lysosomes, i.e., Lamp1-positive vesicles that are not positive for α-synuclein (Figure 6F). This effect was not observed in the presence of BafA1 (Figure 6F). Mature autolysosomes in which the TFL-tag has been degraded could be misclassified as lysosomes, which can potentially explain this effect. In addition, the increased abundance of lysosomes with ML-SA1 can be explained by autolysosome reformation, i.e., the fact that new lysosomes are formed from autolysosomes (Yu et al., 2010; Li et al., 2016). Autolysosome reformation could be stimulated directly by ML-SA1, or potentiated by the increased abundance of autolysosomes (Figure 6D).

TRPML is also known for regulating the intracellular transport of lysosomal vesicles to the cell periphery after starvation (Li et al., 2016). To analyze the possible effects of ML-SA1 on vesicle localization, we performed an analysis of the subcellular distribution of all five particle types (Supplementary Figure 2) as described (Hilverling et al., 2021). We did not observe a significant effect of ML-SA1 on the vesicle localization.

Taken together, these findings with α-synuclein-tagged particles confirm our results with LC3-tagged vesicles (Figure 4) that ML-SA1 reduces the abundance of neutral particles (Figure 5B) and increases the abundance of autolysosomes (Figures 5C,D potentially) and acidic vesicles (Figures 5D,E). The effect of ML-SA1 on the subcellular localization of autophagolysosomal vesicles was not pronounced.

### The Effect of ML-SA1 Could be Partly Based on Lysosome Reformation

Inhibition of mTOR induces autophagy but blocks autolysosome reformation (Yu et al., 2010; Zhang et al., 2016). In order to test the involvement of autolysosome reformation for the ML-SA1 effect, we tested the effect of the mTOR inhibitor rapamycin. As mentioned above, ML-SA1 had no additional effect on EGFP-tagged A53T-α-synuclein in the presence of rapamycin (Figure 4F). Moreover, rapamycin inhibited the effect of ML-SA1 on immature and mature autolysosomes as reported by TFL-tagged A53T-α-synuclein and Lamp1 staining (Figures 5C,D). In particular, the number of lysosomes increased with ML-SA1 in the control condition but decreased with ML-SA1 in the presence of rapamycin (Figure 5E). As one possible explanation, the induction of autophagosome-lysosome fusion by ML-SA1 could deplete lysosomes, in cases where lysosome reformation is inhibited by rapamycin.

### Effect of TRPML1-Agonist MLSA1 and TRPML1-Antagonist ML-SI3 in Neurons

**Figure 7:**
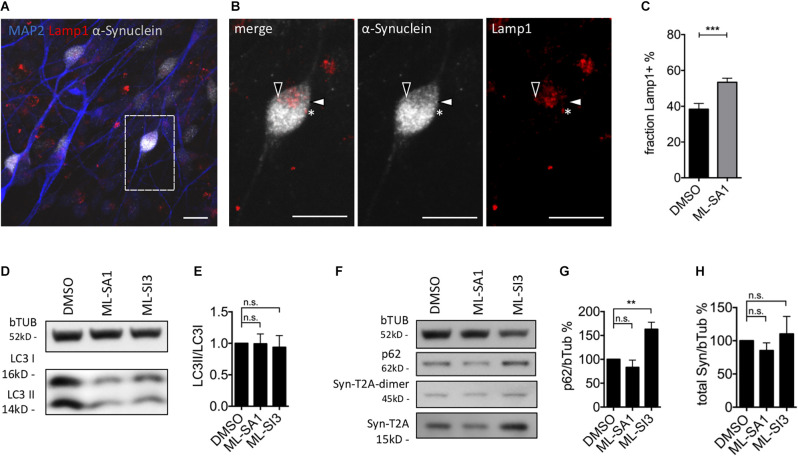
Effect of TRPML1-agonist ML-SA1 and TRPML1-antagonist ML-SI3 on autophagy and α-synuclein particles in human dopaminergic neurons. **(A)** Example images of LUHMES-derived dopaminergic neurons transduced with α-synuclein-T2A-GFP-lentivirus. Six days after transduction neurons were treated for 24 h either with DMSO or 10 μM ML-SA1. Neurons were further stained against human α-synuclein, lysosomal marker Lamp1 and neuronal marker using MAP2. The scale bar represents 20 μm. **(B)** Close up of **(A)**. Closed arrowhead marks a α-synuclein-positive/Lamp1-negative particle, open arrowhead marks a α-synuclein-positive and Lamp1-positive particle, asterisk marks a α-synuclein-negative/Lamp1-positive lysosome. **(C)** Percentage of Lamp1-positive synuclein particles per neuron. Results of 30 cells per condition. Unpaired *t*-test depicted with respect to DMSO (*p* < 0.001). **(D)** Representative immunoblot of lysates from transduced LUHMES-derived neurons on day 9, treated with DMSO, ML-SA1 10 μM, or ML-SI3 10 μM for 2 h. Full blot shown in Supplementary Figure 5A. **(E)** Quantification of the LC3II band relative to LC3I band in *n* = 3 independent experiments as in **(D)**. One-way ANOVA showed no significance (*p* = 0.8). **(F)** Representative immunoblot of lysates from transduced LUHMES-derived neurons on day 9, treated with DMSO, ML-SA1 10 μM or ML-SI3 10 μM for 24 h. Full blot shown in Supplementary Figure 5D. **(G)** Quantification of the p62 band relative to beta-Tubulin in *n* = 3 independent experiments as in **(F)**. One-way ANOVA showed significance (*p* < 0.01). Dunnet’s multiple comparisons test is depicted with respect to DMSO. **(H)** Quantification of Synuclein load relative to beta-Tubulin in *n* = 3 independent experiments as in **(F)**. One-way ANOVA showed no significance (*p* = 0.2). **p < 0.01, ***p < 0.001, non-significant differences are depicted as n.s.

In order to validate the findings in a neuronal cell type, we analyzed the occurrence of Lamp1-positive and Lamp1-negative α-synuclein particles in mature dopaminergic neurons derived from LUHMES cells, which are immortalized human dopaminergic midbrain neurons (Scholz et al., 2011; Neuhof et al., 2021). WT-α-synuclein and GFP were expressed by lentiviral transduction of an aggregate-prone T2A-based construct (Szego et al., 2019). After 1 week of expression, neurons were exposed to ML-SA1 or DMSO for 24 h, its non-toxic concentration was defined by LDH-assay (Supplementary Figure 4C). We stained against human α-synuclein (gray in Figures 7A,B), the lysosomal marker Lamp1 (red in Figures 7A,B), and the neuronal marker MAP2 (blue in Figure 7A). We observed several puncta that were positive for α-synuclein and Lamp1 (e.g., closed arrowheads in Figure 7B), but also puncta that were only positive for synuclein (e.g., open arrowheads in Figure 7B) and puncta only positive for Lamp1 (e.g., asterisk in Figure 7B). We hypothesize that these categories represent autolysosomes, autophagosomes (or cytosolic aggregates), and lysosomes. Neurons treated with ML-SA1 showed a significantly higher percentage of α-synuclein in autolysosomes than in the control condition (Figure 7C).

ML-SI3 is a new compound, found to be an antagonist to TRPML1, but also affects TRPML2 and 3 to some extent (Leser et al., 2021). In order to validate our findings on autophagosome formation (Figures 4, 5) using a different technique, we quantified in lysates of dopaminergic neurons derived from LUHMES cells the fraction of lipidated LC3 (LC3II/LC3I ratio) using immunoblots. Consistent with our findings in HEK293T cells, ML-SA1 and ML-SI3 did not change the amount of lipidated LC3 after 2 h (Figures 7D,E) or 24 h (Supplementary Figures 5A,B). p62 is an autophagic cargo commonly used to quantify autophagic flux (Klionsky et al., 2016). p62 significantly accumulated in the presence of the TRPML inhibitor ML-SI3 (Figures 7F,G), whereas ML-SA1 showed a trend for reduced p62 after 24 h (Figures 7F,G). Yet, administration of ML-SA1 and ML-SI3 for 24 h did not show a significant effect on the amount of overexpressed α-synuclein analyzed in LUHMES cells 9 days after lentivirus-based transduction with α-synuclein-T2A-GFP (Figures 7F–H).

**Figure 8:**
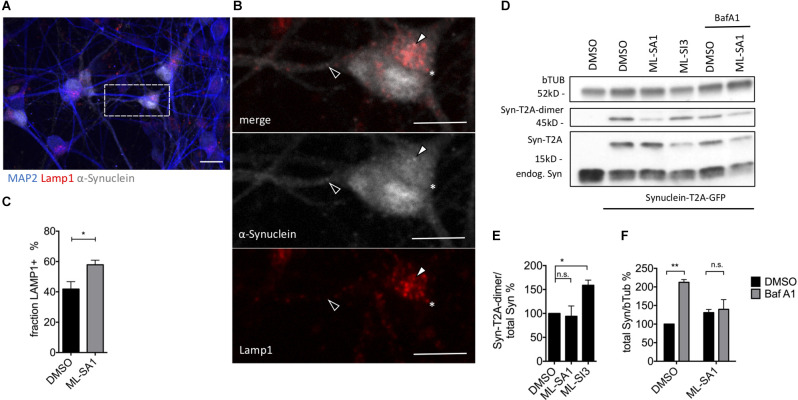
Effect of TRPML1-agonist ML-SA1 and TRPML1-antagonist ML-SI3 on α-synuclein particles in iPSC-derived mature neurons. **(A)** Example images of NPC-derived neurons transduced with α-synuclein-T2A-GFP using lentivirus. Twenty-one days after transduction, cells were treated for 24 h either with 20 μM ML-SA1 or DMSO. Cells were further stained against human α-synuclein, lysosomal marker Lamp1 and neuronal marker MAP2. The scale bar represents 20 μm. **(B)** Close up of **(A)**. Closed arrowhead marks a α-synuclein-positive/Lamp1-negative particle, open arrowhead marks a α-synuclein-positive and Lamp1-positive particle, asterisk marks a α-synuclein-negative/Lamp1-positive lysosome. **(C)** Percentage of Lamp1-positive synuclein particles per cell. Results of at least 30 neurons per condition. Unpaired *t*-test depicted with respect to DMSO (*p* = 0.01). **(D)** Representative immunoblot of lysates from transduced neurons on day 21, treated with DMSO, ML-SA1 10 μM, or ML-SI3 10 μM and additionally DMSO or 100 nM BafA1 for 2 h. Full blot shown in Supplementary Figure 6A. **(E)** Quantification of the Synuclein-T2A-dimer band relative to total synuclein load in *n* = 3 independent experiments as in **(D)**. One-way ANOVA showed significance (*p* = 0.03). Results of Dunnet’s multiple comparisons test are depicted with respect to DMSO (*p* = 0.03). **(F)** Quantification of Synuclein load relative to beta-Tubulin in *n* = 3 independent experiments as in **(D)**. Two-way-ANOVA showed significant interaction (*p* = 0.03), Sidak’s multiple comparisons test is depicted with respect to DMSO. *p < 0.05, **p < 0.01, non-significant differences are depicted as n.s.

In order to check the effect of ML-SA1 and ML-SI3 on α-synuclein in more detail, we used mature neurons that were derived from human-induced pluripotent stem cells (hiPSC). Neurons were analyzed 21 days after lentivirus-based transduction with α-synuclein-T2A-GFP (Figure 8A). By staining for Lamp1, we observed with ML-SA1 a significantly higher percentage of α-synuclein that was packed into autolysosomes (Figures 8B,C). In immunoblot analysis upon ML-SA1 or ML-SI3 treatment, the fraction of lipidated LC3 were not altered (Supplementary Figure 6C). Both findings are consistent with our findings in LUHMES-derived dopaminergic neurons (Figures 7C–E). With respect to α-synuclein, however, we observed a significant increase of aggregate-prone α-synuclein-T2A-dimers with the TRPML1 inhibitor ML-SI3 (Figures 8D,E). The agonist ML-SA1 showed an opposite trend that was not statistically significant. BafA1 increased the total amount of α-synuclein in neurons, and this accumulation was reversed by the TRPML agonist ML-SA1 (Figures 8D–F). The potentiated effect of ML-SA1 in the presence of BafA1 is consistent with TRPML1-induced lysosomal exocytosis of α-synuclein in neurons with inhibited lysosomal degradation (Tsunemi et al., 2019), but also with our findings of enhanced maturation of autophagosomes in the presence of BafA1 in HEK293T cells (Figure 5). Overall, these findings confirm in neurons that TRPML1 promotes autophagosome maturation and increases the abundance of α-synuclein-containing autolysosomal vesicles.

## Discussion

In this work, we confirmed in HEK293T cells that activation of the lysosomal TRPML1 calcium channel by its agonist ML-SA1 reduces α-synuclein load. This effect requires autophagy, the lysosomal signaling lipid PI(3,5)P_2_, and the release of intracellular calcium. In our paradigm, ML-SA1 increased the maturation of autophagosomes into acidic compartments, leading to an increased presence of α-synuclein in acidic vesicles. Consistently, ML-SA1 also promoted the presence of α-synuclein in autolysosomes in human dopaminergic and stem cell derived neurons.

### Reduction of α-Synuclein Aggregates by ML-SA1 Requires Autophagy

ML-SA1 reduced the amount of α-synuclein protein in HEK293T cells (Figure 1H) and the number of visible α-synuclein particles (Figure 1D). This effect was not observed when the initiation of autophagy was blocked by 3-MA or DN Atg5 (Figures 4C,D), blunted when late stages of autophagy were blocked by BafA1 (Figure 4E), and also not observed in the presence of rapamycin (Figure 4F). Of note, rapamycin has diverse cellular effects. The induction of autophagy and the activation of TRPML (Zhang et al., 2019) could contribute to the potential ceiling effect in Figure 4F and the potential floor effect in Figure 5B whereas reduced lysosome reformation (Yu et al., 2010) best explains the effect on lysosomes in Figure 5F. Collectively, the cited findings confirm that ML-SA1 reduces α-synuclein through the auto-lysosomal pathway and not through other mechanisms of α-synuclein clearance such as the proteasome. Accordingly, α-synuclein was observed less frequently in free cytosolic aggregates and more commonly inside vesicles with ML-SA1 in HEK293T cells (Figure 3D). Moreover, α-synuclein was more commonly colocalized in autolysosomes with ML-SA1 in human dopaminergic neurons (Figure 7C) and iPS cell-derived neurons (Figure 8C). The observed reduction of α-synuclein load in HEK293T cells is consistent with the reduction of α-synuclein by ML-SA1 *via* enhanced lysosomal exocytosis in stem cell derived neurons recently observed by others (Tsunemi et al., 2019) and with a large body of literature demonstrating accumulation of α-synuclein with autophago-lysosomal impairment, for instance, caused by the mutation in GBA or deficiency in ATP13A2 (Mazzulli et al., 2011; Usenovic et al., 2012; Schultheis et al., 2013; Lopes da Fonseca et al., 2016). Accordingly, enhanced α-synuclein clearance can be reached by facilitating auto-lysosomal function, for instance by increasing GBA or Rab7 (Dinter et al., 2016; Mazzulli et al., 2016; Saridaki et al., 2018; Szegö et al., 2022).

We assume that ML-SA1 achieved these effects by activation of the TRPML1 cation channel because the effects were not observed in the presence of the calcium chelator BAPTA (Figure 2C) and required the phosphoinositide PI(3,5)P_2_ (Figures 2A,B) that TRPPML1 function depends on (Dong et al., 2010). We note that the ML1N construct has further binding partners than PI(3,5)P_2_ (Hammond et al., 2015) that could underlie the altered baseline with ML1N (Figure 2A), but the lacking effect of ML-SA1 in the presence of ML1N is consistent with ML-SA1 acting through TRPML1. Overall, these findings are consistent with the fact that TRPML mediates calcium release from lysosomes (Grimm et al., 2012; Shen et al., 2012), with the dependence of lysosomal degradation of endosomal cargo on PIKfyve (de Lartigue et al., 2009) and the accumulation of autophagosomal cargo with blockade of Figure 4 and Vac14, which also regulate PI(3,5)P_2_ (Ferguson et al., 2009).

### Mechanism of ML-SA1 in Cells With Intact Auto-Lysosomal Pathway

TRPML1 functions in different lysosome-dependent compartments, including autophagosomes, mitochondrial membrane contact sides, lysosome transport, and lysosome exocytosis (Scotto Rosato et al., 2019; Tsunemi et al., 2019; Peng et al., 2020). Accordingly, different mechanisms have been proposed through which TRPML1 agonists like ML-SA1 produce their effects. For clarity, we discuss here separately the effects of TRPML1 agonists in cells with intact and impaired auto-lysosomal function.

Studying cells with an intact auto-lysosomal system is difficult because there are only a few autophagosomes (Figure 4A). Moreover, the abundance of any vesicle subtype results from a steady state of formation and degradation processes. Autophagosomes are rapidly processed along their maturation pathway, i.e., they fuse with lysosome, acidify, and are reformed into lysosomes. Accordingly, the number of mature autolysosomes—as reported by the TFL tag—is even lower than the number of neutral autophagosomes (Figure 6A). In our hands, 18 h of ML-SA1 increased the number of cells without visible autophagosomes and decreased the number of cells with many autophagosomes (Figure 4B). In particular, the number of neutral autophagosomes decreased (Figure 6B). These findings are best explained by improved autophagosome maturation with ML-SA1 and cannot be explained by the transcriptional activation by TRPML1 of autophagic and lysosomal genes through the transcription factor TFEB (Medina et al., 2015). Accordingly, we did not find an effect of ML-SA1 or ML-SI3 on LC3 lipidation in neurons (Figure 7E, Supplementary Figure 6C, but increased colocalization of α-synuclein with Lamp1; Figures 7C, 8C).

In order to increase the abundance for autophagosomes and reduce their rapid maturation, we used time-lapse microscopy with LC3-TFL cells that were starved and incubated with BafA1. In this paradigm, we observed a continual increase of neutral autophagosomes (Figure 5C) as expected with impaired auto-lysosomal fusion and acidification. ML-SA1 led to an increase in the number of acidic vesicles and to a less pronounced accumulation of neutral autophagosomes (Figures 5C,D). This observation is best explained by improved maturation and thus acidification of autophagosomes, resulting in a reduced accumulation of neutral autophagosomes. The fact that the increase in acidic autolysosomes was transient can be explained by the dwindling number of available neutral precursors and by the fact that degradation of LC3-TFL protein in acidic autolysosomes remained intact.

Based on the TFL-tag alone we cannot discriminate whether ML-SA1 primarily increased autolysosomal fusion or vesicle acidification. Increased autolysosome fusion is consistent with the higher number of vesicles double-stained for LC3 and Lamp1 observed by others (Scotto Rosato et al., 2019) and with the dependence of ML-SA1 on a cytosolic calcium release (Figure 2C), which is a typical trigger for vesicle fusion events mediated by SNARE proteins. For instance, calcium release can promote th fusion of lysosomes with endosomes (Cao et al., 2015) or the plasma membrane (Tsunemi et al., 2019). Depletion of calcium stores by thapsigargin, on the other hand, blocks the fusion of autophagosomes with lysosomes (Ganley et al., 2011; Mauvezin and Neufeld, 2015; Mauvezin et al., 2015). Dependence on calcium release is also consistent with the rapid onset of the ML-SA1 effect observed in time-lapse microscopy (Figure 5D).

### Mechanism of ML-SA1 in Cells With Impaired Auto-Lysosomal Pathway

The effects of improving auto-lysosomal clearance are best observed in a context with impaired auto-lysosomal clearance. Indeed, TRPML1 agonists have been explored as potential therapeutic strategies to overcome auto-lysosomal dysfunction (Chen et al., 2014; Tedeschi et al., 2019; Tsunemi et al., 2019).

In this work, we have focused on the effect of the TRPML agonist ML-SA1 on clearance of α-synuclein in human cells, including dopaminergic neurons and stem cell derived neurons. α-synuclein inhibits autophagosomal turnover by impairing SNAP-29-dependent auto-lysosomal fusion (Tang et al., 2021). In HEK293T cells expressing A53T-α-synuclein, ML-SA1 reduced the abundance of cytosolic α-synuclein aggregates and increased the abundance of α-synuclein in vesicles (Figure 3D). This shift is best explained by increased autophagy of α-synuclein aggregates and is consistent with the observation of increased autophagosome biogenesis with TRPML1 agonists in starved HeLa cells (Scotto Rosato et al., 2019). Yet, increased autophagosome biogenesis cannot explain all the effects of ML-SA1.

In neurons with loss of function mutation in ATP13A2/PARK9, which disrupts lysosomal exocytosis and leads to accumulation of α-synuclein (Tsunemi and Krainc, 2014; Tsunemi et al., 2014), ML-SA1 reduces α-synuclein primarily by facilitating fusion of lysosomes with the plasma membrane, i.e., α-synuclein exocytosis (Tsunemi et al., 2019). We did not assess the effect of ML-SA1 on α-synuclein exocytosis in this study, but enhanced α-synuclein exocytosis cannot explain the higher number of α-synuclein-containing vesicles (Figure 3D), and the shift in vesicle populations (Figure 6).

In our paradigm, ML-SA1 was able to override the block of autolysosome fusion and acidification by BafA1, both in HEK293T cells and in neurons (Figures 4E, 5E, 6, 8H), suggesting that ML-SA1 affects the steps in autophagosome maturation inhibited by BafA1. Accordingly, ML-SA1 produced a shift of α-synuclein-tagged vesicles towards Lamp1-positive vesicles and towards acidic compartments (Figures 5D–F, 7C, 8C). The increase of immature autolysosomes with ML-SA1 in the presence of BafA1 (Figure 6C) indicates that ML-SA stimulates autolysosome fusion, consistent with TRPML being a calcium channel as discussed above. Yet, intracellular calcium not only mediates vesicle fusion but has also been implicated in acidification (Luzio et al., 2007; Morgan et al., 2011). The higher abundance of mature autolysosomes but not immature autolysosomes with ML-SA1 (Figures 6C,D) can be interpreted as evidence for an effect of ML-SA1 on vesicle acidification, but could also result from the abundance of immature autolysosomes being a steady-state of several competing processes. Consistently, phagosome acidification was recently shown to depend on TRPML1 and to be rescued by ML-SA1 in PIKfyve deficient cells (Isobe et al., 2019).

## Conclusion

This work confirms that activating the lysosomal TRPML1 channel can be a beneficial strategy to reduce α-synuclein load in cells. ML-SA1 can improve autophagic clearance by diverse mechanisms. In our paradigm, reducing α-synuclein involves facilitated fusion of autophagosomes with lysosomes upon release of calcium. ML-SA1 was effective in the presence of α-synuclein and able to partially overcome autolysosomal fusion block by BafA1. This indicates that ML-SA1 might also be able to overcome autophagosome maturation defects present in lysosomal storage disorders and neurodegenerative diseases. In PD, autolysosome defects are not restricted to genetic forms as caused by mutations in GBA or ATP13A2 but can also be observed in sporadic PD (Gordevicius et al., 2021). TRPML1 upregulation can also help degrade cholesterol (Wang et al., 2015), suggesting that ML-SA1 could be beneficial in lysosomal storage disorders such as NPC, in which cholesterol accumulates and calcium release from lysosomes is impaired (Shen et al., 2012).

## Data Availability Statement

The original contributions presented in the study are included in the article/Supplementary Material, further inquiries can be directed to the corresponding author.

## Author Contributions

BF and ED conceived research. MP, JB, IR, and ED performed research. NR-M supervised immunoblot techniques. ED, MP, and BF wrote the manuscript. All authors contributed to the article and approved the submitted version.

## Conflict of Interest

The authors declare that the research was conducted in the absence of any commercial or financial relationships that could be construed as a potential conflict of interest.

## Publisher’s Note

All claims expressed in this article are solely those of the authors and do not necessarily represent those of their affiliated organizations, or those of the publisher, the editors and the reviewers. Any product that may be evaluated in this article, or claim that may be made by its manufacturer, is not guaranteed or endorsed by the publisher.

## Supplementary Material

Supplementary Figure 1 **(A)** Characteristic examples of GFP fluorescence distribution patterns in HEK293T cells. Fluorescent puncta (small arrowheads) were termed particles. Cells showing no puncta were counted as “homogenous distribution” and cells with puncta as punctate distribution. Cells containing single, large, perinuclear fluorescent puncta were termed as cells with aggresome (large arrowhead) based on previous findings (Opazo et al., 2008). Unhealthy cells were characterized by a rounded appearance. The scale bar represents 10 μm. **(B)** To determine the highest non-toxic concentration of the selective Ca^2+^ chelator BAPTA-AM, we treated HEK293T cells with concentrations of 0.1 μM, 1.0 μM, 10 μM, and 100 μM for 18 h. We counted cells that showed an unhealthy rounded appearance, referred as to be preapoptotic. One-way ANOVA was significant (*p* < 0.0001). Dunnet’s multiple comparisons test is depicted with respect to Control (DMSO). **(C)** Full immunoblot of bands shown in Figure 1G. Blots were cut at 37kD, upper parts were incubated with anti-actin-, lower part with anti-α-synuclein-antibody. As described previously, incubation with the α-synuclein antibody in HEK293T lysates showed an unspecific band at around 35 kDa and two bands around 20 kDa. The upper one was only seen in cells transfected with α-synuclein, and disappeared under synuclein-siRNA treatment and is, therefore, considered as the α-synuclein band (Dinter et al., 2016).

Supplementary Figure 2 Shrink analysis of particles shown in Figure 3 with 10% representing the most central 10% of the cell and 100% representing the entire cell (see methods in Hilverling et al. (2021). HEK293T cells were transfected with A53T-α-synuclein fused to mCherry-FRB and co-expressed with EGFP-FKBP. Addition of rapamycin FKBP leads to heterodimerization of FRB and FKBP, recruiting GFP-FKBP to cytosolic synuclein-mCherry-FRB but not to synuclein contained in vesicles. Six hours after transfection, cells were treated for 18 h either with 25 μM ML-SA1 or DMSO. Distribution of neutral and acidic vesicles from *n* = 3 independent experiments. **(A)** Cumulative probability distribution of cytosolic particles (yellow). Two-way ANOVA (factors treatment and cellring) showed no significant interaction (*p* > 0.9999). **(B)** Cumulative probability distribution of vesicles (red). Two-way ANOVA (factors treatment and cellring) showed no significant interaction (*p* > 0.9999).

Supplementary Figure 3 Shrink analysis of particles shown in Figure 6. HEK293T cells transfected with A53T-α-synuclein tagged mRFP-EGFP tandem-fluorescence (TFL) and treated for 18 h either with 25 μM ML-SA1 or DMSO and additionally 5 nM Bafilomycin A1 or 100 nM Rapamycin or DMSO as control. Cells were further stained against the lysosomal marker Lamp1. **(A–E)** Cumulative probability distribution of particles in presence of DMSO, ML-SA1, BafA1, and ML-SA1 together with BafA1. Two-way ANOVA for all vesicle subtypes (factors treatment and cellring) showed no significant interaction.

Supplementary Figure 4 LUHMES cell differentiation and compound toxicity test. **(A)** Representative images of undifferentiated (day 0) and differentiated (day 9) LUHMES cells, stained for proliferation marker Nestin, Ki-67 and mature neuronal marker beta-Tubulin III (TUBB3) and Map2. Nuclear staining with DAPI. The scale bar represents 50 μm. **(B)** Representative images of differentiated (day 9) LUHMES cells, stained for dopaminergic markers Thyrosinhydroxylase (TH), vesicular monoaminase transporter 2 (VMAT2), and endogenous α-synuclein. Nuclear staining with DAPI. The scale bar represents 50 μm. **(C)** Results of LDH-assay of differentiated LUHMES cells (day 9) treated with DMSO, MLSA1, or ML-SI3 in depicted concentrations for 24 h. Two-way ANOVA showed significant interaction for treatment and concentration (*p* = 0.0023). Dunnet’s multiple comparisons test is depicted with respect to DMSO.

Supplementary Figure 5 Immunoblot analysis in luhmes. **(A)** Full immunoblot of bands shown in Figure 7D. PVDF membrane was cut at 35 kD, the lower part was incubated with anti-LC3 antibody and the upper part was incubated with anti-beta-Tubulin antibody. Secondary antibody and development was performed as described in the method section. **(B)** Representative full immunoblot of lysates from transduced LUHMES day 9, treated with ML-SA1 10 μM, ML-SI3,10 μM or DMSO for 24 h. **(C)** Quantification of the LC3II band relative to LC3I band in *n* = 3 independent experiments as in **(B)**. One-way ANOVA showed no significance (*p* = 0.3). **(D)** Full immunoblot of bands shown in Figure 7F. Nitrocellulose membrane was first incubated with anti-α-synuclein antibody and sequentially labeled with HRP-antibody, followed by detection. Note the weak expression of endogenous α-synuclein (14 kD), labeled with an asterisk. The second band marks the overexpressed and cleaved lentiviral product Synuclein-T2A (22kD). The upper band shows overexpressed dimer-protein of synuclein-T2A (45kD). Afterward, anti-p62-antibody and anti-beta-tubulin-antibody were detected separately on the same membrane.

Supplementary Figure 6 Immunoblot analysis in iPSC-derived neurons. **(A)** Full immunoblot of bands shown in Figure 8D. The nitrocellulose membrane was first incubated with anti-α-synuclein antibody and sequentially labeled with HRP-antibody, followed by detection. Afterwards, anti-beta-tubulin was detected on the same membrane. Mention the band for endogenous a-synuclein at around 14 kD. The second band marks the overexpressed and cleaved lentiviral product synuclein-T2A (22 kD). Upper band shows overexpressed dimer-protein of synuclein-T2A (45 kD). **(B)** Representative full immunoblot of lysates from transduced hiPSC-derived neurons, treated with ML-SA1 10 μM, ML-SI3 10 μM, or DMSO for 2 h. PVDF membrane was cut at 35 kD, lower part was incubated with anti-LC3 antibody, and the upper part was incubated with anti-beta-tubulin antibody. **(C)** Quantification of the LC3II band relative to LC3I band in *n* = 3 independent experiments as in **(B)**. One-way ANOVA showed no significance (*p* = 0.372).
