# Supplementary material for: Activated Endolysosomal Cation Channel TRPML1 Facilitates Maturation of α-Synuclein-Containing Autophagosomes
Source: Front Cell Neurosci. 2022 Jul 6;16:861202. doi: 10.3389/fncel.2022.861202 (PMC9296810; doi:10.3389/fncel.2022.861202)
Supplement: Supplementary Figure 1 — (A) Characteristic examples of GFP fluorescence distribution patterns in HEK293T cells. Fluorescent puncta (small arrowheads) were termed particles. Cells showing no puncta were counted as “homogenous distribution” and cells with puncta as punctate distribution. Cells containing single, large, perinuclear fluorescent puncta were termed as cells with aggresome (large arrowhead) based on previous findings (Opazo et al., 2008). Unhealthy cells were characterized by a rounded appearance. The scale bar represents 10 μm. (B) To determine the highest non-toxic concentration of the selective Ca2+ chelator BAPTA-AM, we treated HEK293T cells with concentrations of 0.1 μM, 1.0 μM, 10 μM, and 100 μM for 18 h. We counted cells that showed an unhealthy rounded appearance, referred as to be preapoptotic. One-way ANOVA was significant (p < 0.0001). Dunnet’s multiple comparisons test is depicted with respect to Control (DMSO). (C) Full immunoblot of bands shown in Figure 1G. Blots were cut at 37kD, upper parts were incubated with anti-actin-, lower part with anti-α-synuclein-antibody. As described previously, incubation with the α-synuclein antibody in HEK293T lysates showed an unspecific band at around 35 kDa and two bands around 20 kDa. The upper one was only seen in cells transfected with α-synuclein, and disappeared under synuclein-siRNA treatment and is, therefore, considered as the α-synuclein band (Dinter et al., 2016). [file Data_Sheet_1.pdf]

Supplementary Material

Activated Endolysosomal Cation Channel TRPML1 Facilitates Maturation of α-Synuclein-Containing Autophagosomes

Maike R. Pollmanns[1], Judith Beer[2], Ines Rosignol[3,4], Natalia Rodriguez-Muela[3,4,5], Björn H. Falkenburger[1,2,3,6]* and Elisabeth Dinter[1,2,3]
[1] Department of Neurology, RWTH University Aachen, Aachen, Germany, [2]Department of Neurology, University Hospital Carl Gustav Carus, Technische Universität Dresden, Dresden, Germany, [3]Deutsches Zentrum für Neurodegenerative Erkrankungen (DZNE), Dresden, Germany, [4]Center for Regenerative Therapies Dresden (CRTD), Dresden, Germany, [5]Max Planck Institute for Molecular Cell Biology and Genetics, Dresden, Germany, [6]JARA-Institute Molecular Neuroscience and Neuroimaging, Forschungsszentrum Jülich GmbH and RWTH Aachen University, Aachen, Germany

Supplementary Figure 1

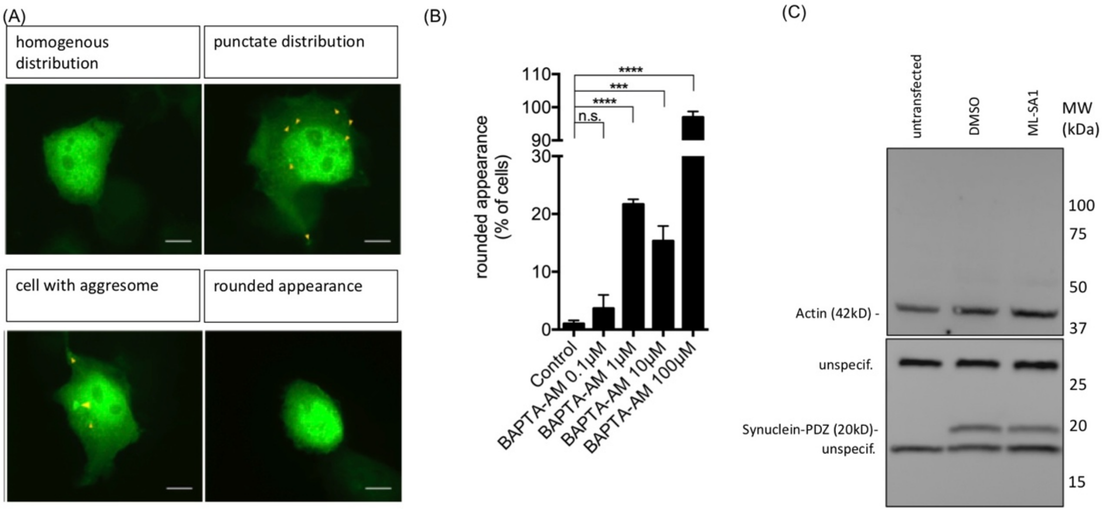

(A) Characteristic examples of GFP fluorescence distribution patterns in HEK293T cells. Fluorescent puncta (small arrowheads) were termed particles. Cells showing no puncta were counted as “homogenous distribution” and cells with puncta as punctate distribution. Cells containing single, large, perinuclear fluorescent puncta were termed as cells with aggresome (large arrowhead) based on previous findings (Opazo et al., 2008). Unhealthy cells were characterized by a rounded appearance. Scale bar represents 10 µm. **(B)** To determine the highest non-toxic concentration of the selective Ca2+ chelator BAPTA-AM, we treated HEK293T cells with concentrations of 0.1 µM, 1.0 µM, 10µM and 100 µM for 18 hours. We counted cells that showed unhealthy rounded appearance, referred as to be preapoptotic. One-way ANOVA was significant ($p<0.0001$). Dunnet’s multiple comparisons test is depicted with respect to Control (DMSO). **(C)** Full immunoblot of bands shown in **Figure 1G**. Blots were cut at 37kD, upper parts were incubated with anti-actin-, lower part with anti-α-synuclein-antibody. As described previously, incubation with the α-synuclein antibody in HEK293T lysates showed an unspecific band at around 35 kDa and two bands around 20 kDa. The upper one was only seen in cells transfected with α-synuclein, disappeared under synuclein-siRNA treatment and is, therefore, considered as the α-synuclein band (Dinter et al., 2016).

Supplementary Figure 2

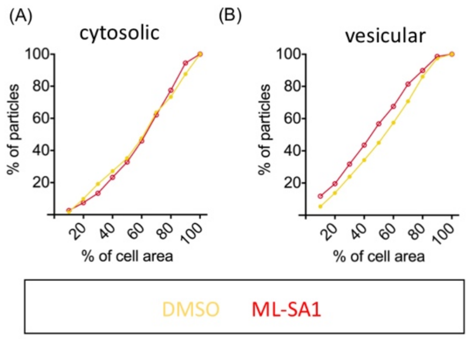

Shrink analysis of particles shown in **Figure 3** with 10% representing the most central 10% of the cell and 100% representing the entire cell (see methods in (Hilverling et al., 2021)): HEK293T cells were transfected with A53T-α-synuclein fused to mCherry-FRB and co-expressed with EGFP-FKBP. Addition of rapamycin FKBP leads to heterodimerization of FRB and FKBP, recruiting GFP-FKBP to cytosolic synuclein-mCherry-FRB but not to synuclein contained in vesicles. 6h after transfection, cells were treated for 18 hours either with 25 µM ML-SA1 or DMSO. Distribution of neutral and acidic vesicles from n=3 independent experiments **(A)** Cumulative probability distribution of cytosolic particles (yellow). Two-way ANOVA (factors treatment and cell ring) showed no significant interaction ($p > 0.9999$). **(B)** Cumulative probability distribution of vesicles (red). Two-way ANOVA (factors treatment and cell ring) showed no significant interaction ($p > 0.9999$).

Supplementary Figure 3

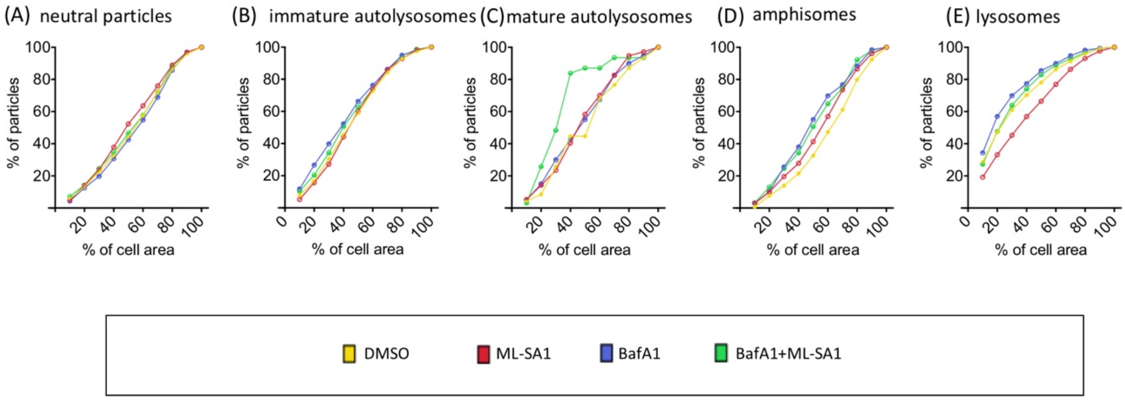

Shrink analysis of particles shown in **Figure 6**. HEK293T cells transfected with A53T-α-synuclein tagged mRFP-EGFP tandem-fluorescence (TFL) and treated for 18 hours either with 25 µM ML-SA1 or DMSO and additionally 5nM Bafilomycin A1 or 100nM Rapamycin or DMSO as control. Cells were further stained against the lysosomal marker Lamp1. **(A)-(E)** Cumulative probability distribution of particles in presence of DMSO, ML-SA1, BafA1 and ML-SA1 together with BafA1. Two-way ANOVA for all vesicle subtypes (factors treatment and cell ring) showed no significant interaction.

Supplementary Figure 4

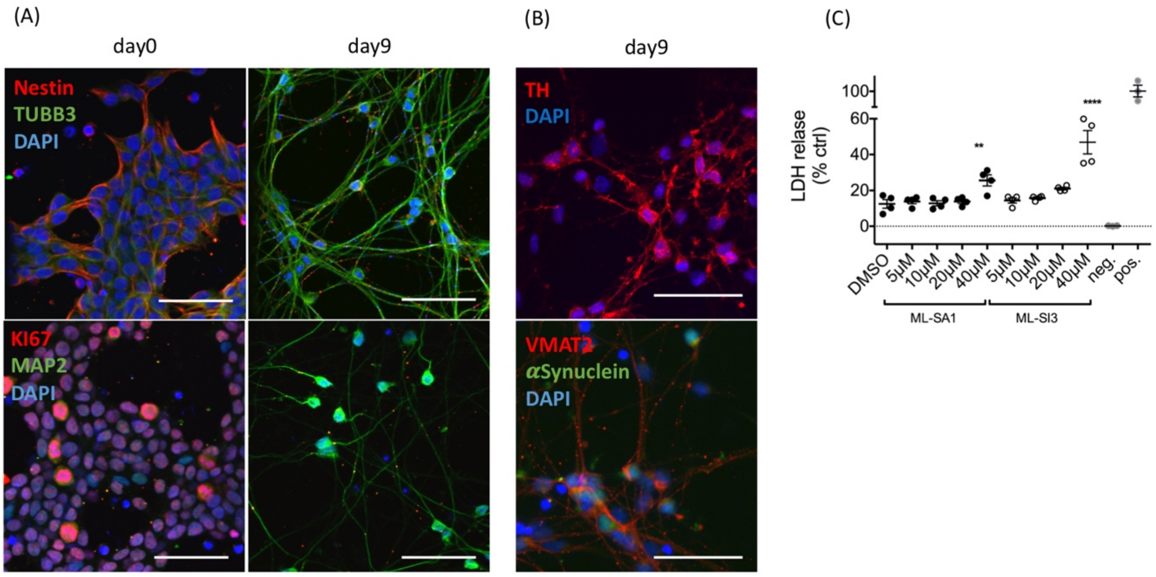

LUHMES cell differentiation and compound toxicity test. **(A)** Representative images of undifferentiated (day 0) and differentiates (day 9) LUHMES cells, stained for proliferation marker Nestin, Ki-67 and mature neuronal marker beta-Tubulin III (TUBB3) and Map2. Nuclear staining with DAPI. Scale bar represents 50 µm. **(B)** Representative images of differentiated (day 9) LUHMES cells, stained for dopaminergic markers Thyrosinhydroxylase (TH), vesicular monoaminase transporter 2 (VMAT2) and endogenous α-synuclein. Nuclear staining with DAPI. Scale bar represents 50 µm **(C)** Results of LDH-assay of differentiated LUHMES cells (day 9) treated with DMSO, MLSA1 or ML-SI3 in depicted concentrations for 24 hours. Two-way ANOVA showed significant interaction for treatment and concentration ($p = 0.0023$). Dunnet's multiple comparisons test is depicted with respect to DMSO.

Supplementary Figure 5

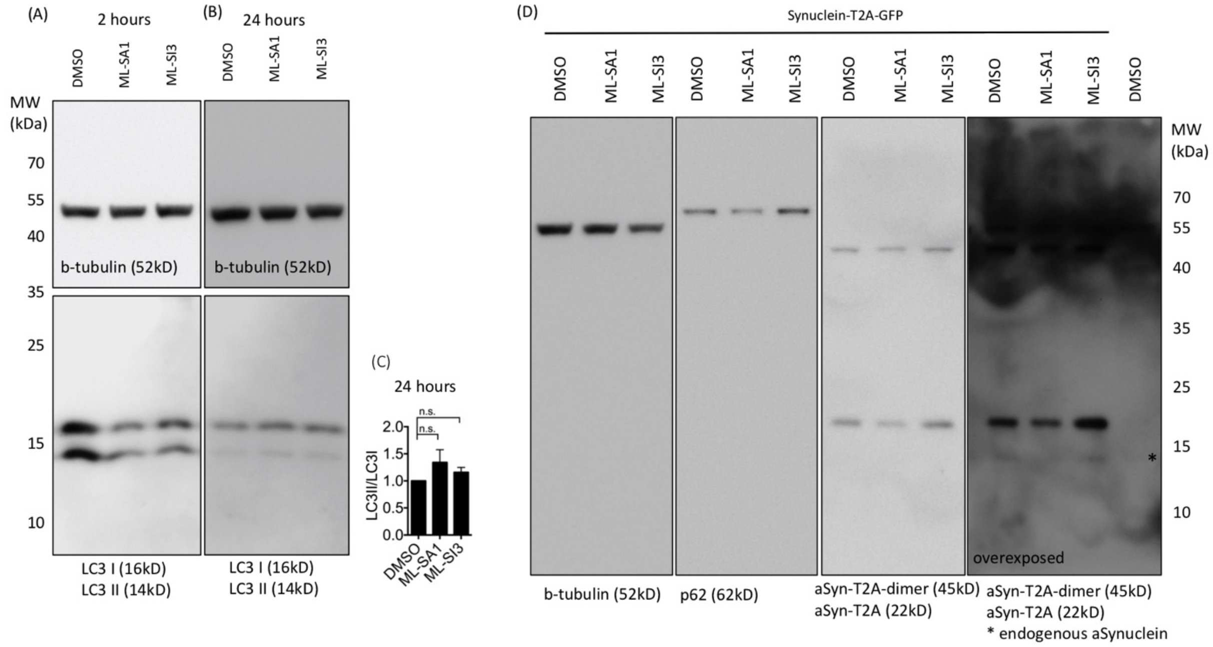

Immunoblot analysis in LUHMES **(A)** full immunoblot of bands shown in **Figure 7D**. PVDF membrane was cut at 35kD, lower part was incubated with anti-LC3 antibody, upper part was incubated with anti-beta-Tubulin antibody. Secondary antibody and development was performed as described in the method section. **(B)** Representative full immunoblot of lysates from transduced LUHMES day 9, treated with ML-SA1 10 µM, ML-SI3 10 µM or DMSO for 24 hours. **(C)** Quantification of the LC3II band relative to LC3I band in n=3 independent experiments as in **(B)**. One-way ANOVA showed no significance ($p=0.3$). (D) Full immunoblot of bands shown in **Figure 7F**. Nitrocellulose membrane was first incubated with anti-α-synuclein antibody and sequentially labelled with HRP-antibody, followed by detection. Note the weak expression of endogenous α-synuclein (14 kD), labelled with asterisk. Second band marks the overexpressed and cleaved lentiviral product Synuclein-T2A (22kD). Upper band shows overexpressed dimer-protein of Synuclein-T2A (45kD). Afterwards, anti-p62-antibody and anti-beta-Tubulin-antibody were detected separately on the same membrane.

Supplementary Figure 6

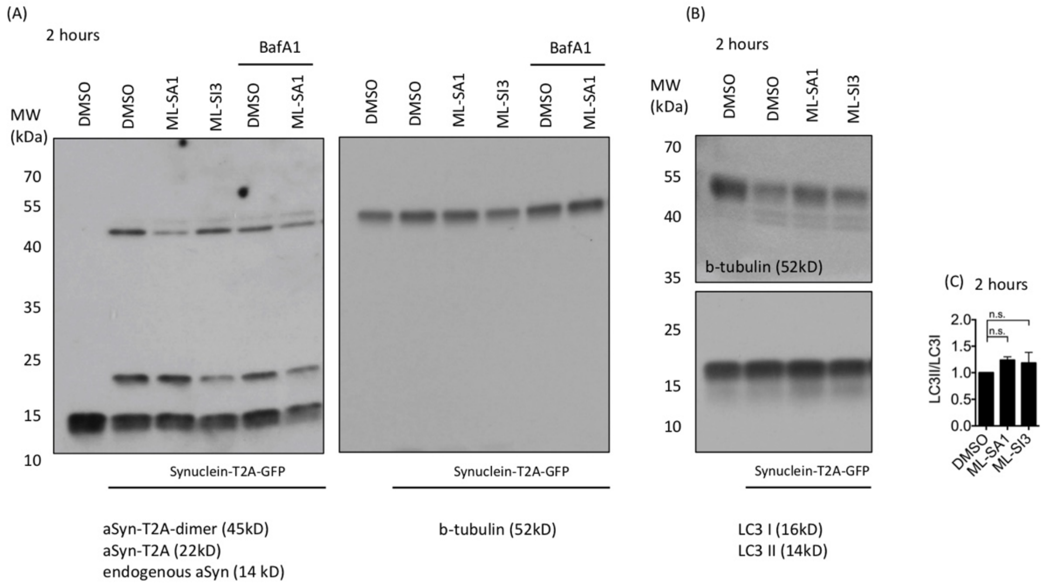

Immunoblot analysis in iPSC-derived neurons. **(A)** Full immunoblot of bands shown in **Figure 8D**. Nitrocellulose membrane was first incubated with anti-α-synuclein antibody and sequentially labelled with HRP-antibody, followed by detection. Afterwards, anti-beta-Tubulin was detected on the same membrane. Mention the band for endogenous α-Synuclein at around 14kD. Second band marks the overexpressed and cleaved lentiviral product Synuclein-T2A (22kD). Upper band shows overexpressed dimer-protein of Synuclein-T2A (45kD). **(B)** Representative full immunoblot of lysates from transduced hiPSC-derived neurons, treated with ML-SA1 10 µM, ML-SI3 10 µM or DMSO for 2 hours. PVDF membrane was cut at 35kD, lower part was incubated with anti-LC3 antibody, upper part was incubated with anti-beta-Tubulin antibody. **(C)** Quantification of the LC3II band relative to LC3I band in n=3 independent experiments as in **(B)**. One-way ANOVA showed no significance (p=0.372)
